# The antiprotease Spink7 promotes inflammation resolution by modulating multiple proteases activities during wound healing

**DOI:** 10.1002/ctm2.70291

**Published:** 2025-03-27

**Authors:** Na Zhao, Guojian Wang, Shuang Long, Xiaofan Lv, Xinze Ran, Junping Wang, Yongping Su, Tao Wang

**Affiliations:** ^1^ Institute of Combined Injury Chongqing Engineering Research Center for Nanomedicine School of Preventive Military Medicine Army Medical University (Third Military Medical University) Chongqing China; ^2^ Institute of Materia Medica and Department of Pharmaceutics College of Pharmacy Army Medical University (Third Military Medical University) Chongqing China; ^3^ State Key Laboratory of Trauma and Chemical Poisoning Chongqing China

**Keywords:** inflammation resolution, proteases, radiation‐wound combined injury, Spink7, wound healing

## Abstract

**Background:**

Effective control of inflammation is crucial for the healing of cutaneous wounds, but the molecular mechanisms governing inflammation resolution during wound closure are still not yet clear. Here, we describe a homeostatic mechanism that facilitates the inflammation resolution by timely regulating the targeted proteases activities through antiprotease Spink7 (serine peptidase inhibitor, kazal type 7).

**Methods:**

The expression pattern of Spink7 was investigated by quantitative RT‐PCR, immunohistochemistry (IHC) and in situ hybridization. In both Spink7 knockdown and knockout models, quantitative comparisons were made between the healing rate of wounds and histopathological morphometric analysis. Microarrays, multiple chemokine assays, IHC, immunofluorescence, protease activity measurement were performed to explore the underlying mechanisms of Spink7 knockout in impaired wound healing. Radiation‐wound combined injury (R‐W‐CI) model was employed to evaluate the therapeutic effects of Spink7 manipulation.

**Results:**

Our study demonstrates that Spink7 is significantly upregulated in the differentiated epidermal granular keratinocytes of proliferative phase during murine wound closure. Both local knockdown of Spink7 levels in wounds using siRNA gel and systemic knockout of Spink7 using KO mice resulted in delayed wound closure with sustained neutrophil infiltration. Loss of Spink7 leads to augmented inflammatory responses, increased production of multiple chemokines/cytokines, and impaired M2 polarization of macrophages in wound healing. Furthermore, loss of Spink7 results in elevated proteolytic activities of uPA, MMP2/9 and KLK5/7 in proliferative phase. However, inhibiting KLK5/7 downstream PAR2 activation exacerbates the phenotype of KO mice. In R‐W‐CI model, further significant induction of Spink7 is observed in wounds with insufficient inflammatory response. Local suppression of Spink7 promotes wound healing in the R‐W‐CI model by augmenting inflammation.

**Conclusions:**

Maintaining an endogenous balance between Spink7 and its target proteases is a crucial checkpoint for regulating inflammation resolution during healing. Therefore, manipulating levels of Spink7 might be an effective treatment for impaired wounds caused by inflammatory dysregulation.

## Graphical abstract

• Spink7, which originates from differentiated epidermal granular keratinocytes of proliferative phase, plays a crucial role during skin wound healing.

• Spink7 promotes inflammation resolution by suppressing multiple proteases activities including uPA, MMP2/9, and KLK5/7 during skin wound healing.

• Spink7 promotes the transition from inflammation to proliferation through inhibiting production of cytokines/chemokines and modulating M2 polarization of macrophage.

• Modulating Spink7 levels by siRNAs in wounds of radiation combined injury facilitates healing by enhancing inflammation.

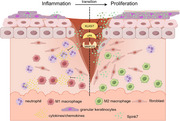

## 1 INTRODUCTION

Wound healing is one fundamental physiological process which maintains the integrity of skin through a series of well‐orchestrated biological and molecular events.^1^, ^2^ The whole cutaneous wound healing process includes three distinct, yet sequential and overlapping phases: inflammatory phase, proliferative phase, and remodelling phase. The initial stage is inflammation, which is indispensable to clear invading pathogens and tissue debris. The subsequent proliferative phase is characterized by re‐epithelialization, formation of granulation tissue, angiogenesis and matrix deposition. The wound healing ends with scar formation or regeneration in the remodelling phase.^3^, ^4^, ^5^ During the process, an appropriate transition from one phase to the next is pivotal for proper healing. Defects of transitions between contiguous phases may result in delayed wound healing, excessive scarring, or chronic wounds. In particular, the transition from the inflammatory to the proliferative phase is one critical control point and determines the outcome of wound healing.^4^

Proper resolution of inflammation is the key event during the inflammation‐proliferation transition, which is tightly controlled and facilitates wound healing.^6^, ^7^, ^8^, ^9^ Otherwise, excessive and persistent inflammation leads to the failure to enter the proliferative phase, which might result in chronic non‐healing wounds. Conversely, if the inflammation resolves prematurely, or if the inflammatory response is inadequate, it can also lead to delayed healing due to insufficient stimulation of the repair cells. A typical instance is the wound of radiation combined injury (RCI). RCI refers to special circumstances where radiation injury is coupled simultaneously or successively with other insults such as burns or wounds.^10^, ^11^, ^12^ Owing to hematopoietic and immune system suppression caused by ionizing radiation injury, the wounds of RCI present delayed healing phenomenon due to insufficient inflammatory response.^12^, ^13^, ^14^ There will be mass victims of RCI in serious nuclear accidents, which would be the focus of public health concern. However, there is still a lack of effective means for wound treatment of RCI. We believe that clarifying the mechanism of inflammation resolution in wound healing might also offer new ideas for the management of RCI wounds.

Extensive works have revealed that resolution of inflammation is a carefully managed active process involving apoptosis of neutrophils, efferocytosis, polarization of macrophages, regulation of cytokines, and so on.^6^, ^7^, ^8^, ^9^ Additionally, the characteristics of inflammatory responses in epidermal cells and their role in the inflammation resolution during wound repair have drawn growing attention recently.^15^, ^16^ However, there are few studies on the role of proteases and their inhibitors in inflammation resolution, although it is generally believed that their tight regulation is important for proper inflammation resolution and even wound healing. Previous studies suggest that uncontrolled proteases activities are common features of chronic wounds.^1^, ^17^, ^18^, ^19^ And there is a significant upregulated in serine proteases in chronic wounds, which results in over degradation of matrix components and trapped into persistent proinflammatory state.^20^, ^21^, ^22^, ^23^ Hence, it is of great significance to explore the effect of endogenous protease inhibitors on the resolution of inflammation and to clarify their role in wound healing.

The serine protease inhibitor Kazal type (SPINK) family is the largest branch in the serine protease inhibitor family, which prevents the imbalance of protease activity by regulating serine proteases. Previous studies have shown that some SPINKs are mainly expressed in the skin and are implicated in the maintenance of skin function and the occurrence of some skin diseases.^24^, ^25^, ^26^ However, their function in wound healing, particularly in the regulation of inflammation resolution, remains poorly understood. SPINK7, also referred to as oesophageal cancer‐related gene 2 (*ECRG2*), is a novel molecule possessing inflammatory regulatory functions.^27^, ^28^, ^29^ Its role in skin tissue remains unknown and is worthy of exploration. SPINK7 was initially identified as tumour suppressor of oesophageal cancer that suppresses tumour cell migration and invasion through inhibiting uPA/uPAR signalling.^30^, ^31^, ^32^, ^33^, ^34^ Further studies indicate that DNA damage response induces upregulation of SPINK7 through p53 signalling pathway, which results in promoting caspase 3 activation and cell apoptosis.^35^, ^36^, ^37^ Previously, the novel role of SPINK7 as an inhibitory checkpoint for inflammatory response was revealed.^27^, ^28^ SPINK7 deficiency results in the unleashing of proteolytic activities and proinflammatory innate responses in oesophageal epithelium through regulating uPA and kallikrein 5 (KLK5), which makes significant contributions to eosinophilic esophagitis (EoE) pathogenesis.^27^, ^28^ What's more, our recent work indicates that Spink7 derived from neutrophils exerts a protective effect by inhibiting multiple proinflammatory cytokines and chemokines in experimental murine colitis models.^29^ Previous report shows that expression of SPINK7 increases in psoriasis and eczema.^38^ However, the role of SPINK7 in cutaneous disorders, especially skin injuries, is still not clear.

Here, we show that Spink7 is markedly induced upregulation in the differentiated epidermal granular keratinocytes during the proliferative phase of murine wound repair. Functional studies indicate local silencing of Spink7 through siRNA gel and systematic knockout Spink7 using KO mice result in delayed wound closure characterized by excessive inflammation with enhanced chemokines/cytokines. Further investigation shows that Spink7 deficiency enhances neutrophils infiltration and affects macrophages M2 polarization in proliferative phase. What's more, loss of Spink7 leads to augment proteolytic activities of uPA, MMP2/9, and kallikreins (KLKs). To assess the translational potential of targeting Spink7 for promoting wound healing, a classical RCI model, radiation‐wound combined injury (R‐W‐CI), is employed. And the findings suggest that knockdown Spink7 by siRNA gel in wounds of R‐W‐CI promotes healing through augmenting inflammation responses. In conclusion, we have identified an endogenous, homeostatic mechanism for inflammation resolution during cutaneous wound healing and indicated that balance between Spink7 and its multiple target proteases is the key for proper wound repair.

## 2 METHODS AND MATERIALS

### 2.1 Animals

All experiments related to mice have been approved by the Laboratory Animal Welfare and Ethics Committee of the Army Medical University (AMU). Spink7 knockout (KO) mice (S‐KO‐10030) were purchased from Cyagen Biotechnology.^29^ C57BL/6J mice were obtained from SiPeiFu Biotechnology. All the mice were bred and raised at the Experimental Animal Center, AMU.

### 2.2 Wound healing studies

The skin wound healing model has been described previously.^39^ In brief, the C57BL/6J mice aged 6–8 weeks were anaesthetized, and their dorsal area was shaved. Then, two 6.0 mm wounds were generated with a sterile punch. At certain time points, mice were euthanized, and wound tissues were harvested with an 8.0‐mm biopsy punch for either RNA isolation, protein extraction, or histopathological analysis.

Two siRNAs against Spink7 were designed and synthesized from GenePharma to knockdown Spink7 in the wound sites. The target sequences were as follows: si‐Spink7‐61: GAAGTTACTAGCCACCCTT; si‐Spink7‐160: GGATCTGACTATATCACTT. Firstly, a 30% concentration of Pluronic F‐127 (P2443, Sigma) gel was fabricated by dissolving PBS, which was liquid at low temperature (4°C). Subsequently, the siRNAs were mixed into the gel at a concentration of 5 µM under ice bath conditions. And the F127 gel containing siRNAs was applied immediately following wound creation with 60 µL siRNA gel per wound as described previously.^40^ To assess the impact of Spink7 knockout (KO) on wound healing, wounds were created on the back skin of both Spink7 KO and wild‐type (WT) littermates. Therefore, the wound healing process was digitally photographed at different time points and residual wound area measurement was performed using ImageJ (version 2.1.4.6, NIH) as reported previously.^39^

To explore the function of PAR2 activation in impaired wound healing of Spink7 KO mice, the treatment of specific antagonist ENMD‐1068 (ab141699, Abcam) was performed in murine wound healing in vivo model. ENMD‐1068 was administrated every day from ‐1 to 6 day after wounding with dosage of 10 mg/kg by intraperitoneal injection. Twenty‐four hours after the last injection, skin wound samples were taken for either protein extraction or RNA isolation.

### 2.3 Radiation‐wound combined injury (R‐W‐CI) studies

R‐W‐CI model has been reported previously with modification.^41^ Briefly, the C57BL/6J mice ranging from 6 to 8 weeks old were subjected to total body irradiation with a single dose of 6.0 Gy by ^60^Co γ‐ray at dose rate 0.649 Gy/min. And then, as mentioned above, two 6.0 mm circular wounds were generated within 1 h after irradiation. For microarray experiments, the wounds of Day 3 after wounding from R‐W‐CI mice and wounded‐only mice were taken. The siRNAs gel targeting Spink7 was employed to assess the effects of Spink7 knockdown at topical wound sites on wound healing of R‐W‐CI, and it was smeared into wound with a single volume of 60 µL in R‐W‐CI model.

### 2.4 Quantitative real‐time PCR

Total RNA was extracted by the RNAiso Plus (9109, TaKaRa) according to the manufacturer's protocol. Quantitative real‐time PCR (qRT‐PCR) was conducted on a Bio‐Rad CFX Connect Real‐Time System with SYBR Premix (RR820A, TaKaRa). The ddCt method was performed for data analysis and the results were normalized to TBP (TATA binding protein) or GAPDH expression. The primer sequences used are listed in Table S1.

### 2.5 Costaining RNA fluorescence in situ hybridization and immunofluorescence

RNA fluorescence in situ hybridization (RNA FISH) and immunofluorescence (IF) were performed on 4 µm formalin fixed paraffin‐embedded (FFPE) sections obtained from skin wound samples. Sections were pre‐treated with target retrieval reagents and protease to improve target recovery based on protocol of the RNAScope Multiplex Fluorescent Detection Kit v2 (cat. no. 323100, ACDBio) as previously reported.^42^ Probe against mouse Spink7 (cat. no. 1191121‐C1, ACDBio) mRNA molecule was used. Samples were then stained for Ly6G (1:100, Biolegend) or E‐cadherin (1:400, CST) for 3‐day or 7‐day wounds sections respectively to label neutrophils or keratinocytes of wound tissues using the AF488‐conjugated secondary antibodies. Tissues were counterstained with DAPI. Images were taken with TCS SP5 confocal microscope (Leica Microsystems) using 40× oil immersion lens.

### 2.6 Immunohistochemistry and immunofluorescence

Immunohistochemistry (IHC) and immunofluorescence (IF) staining were performed following the exact procedures and protocols as previously described.^29^ For IHC, skin wound tissue sections were stained with customized rabbit anti‐Spink7 (1:400, custom antibody generated by immunizing rabbits with synthesized peptide TEILRSNGKIQFLHEGHC corresponding to the C‐terminus of Spink7 through SinoBiological) and commercialized primary antibodies for MPO (myeloperoxidase), uPA, MMP2, MMP9, KLK5, and KLK7 respectively. Images were captured by Olympus IX73‐A21PH microscope (Olympus, Japan) after DAB staining. For IF, the sections were incubated with rat anti‐F4/80, rabbit anti‐iNOS and rabbit anti‐CD206 overnight at 4°C. Then, the sections were incubated for 1 h at RT with Alexa Fluor 488 goat anti‐rabbit or Aleca Fluor 594 goat anti‐rat immunoglobulin G (H+L) (Lifetech). Detailed information of the primary and secondary antibodies utilized for these analyses are provided in Table S2A. The sections were then stained with DAPI followed by covering sections. Images were captured with ZEISS LSM800 confocal microscopy. Quantifications for MPO, F4/80, CD206, iNOS, MMP2, and MMP9 were carried out through using Image Pro Plus software by calculating the percentage of positive cells with 6–8 40× high power field consecutive photos per mouse. Qualitative analyses of KLK5 and KLK7 were carried out by the proportion of the area occupied by positive signals with 3–5 20× high power field photos per mouse.

### 2.7 Histopathological and morphometric analysis

Serial sections of wound tissues were cut from wound midline and H&E stained. The largest cross section of each wound was used for histopathological measurements and morphometric analysis as reported previously.^39^ Wound width was measured as the distance between the wound margins defined by the last hair follicles. Epidermal length was determined as the length of migrating tongue from the site of epidermis with broken dermis to the distal end. The percentage of re‐epithelialization was calculated as distance covered by epithelium dividing the wound width. Wound area was calculated as the area of granulation tissue.

### 2.8 RNA microarrays and data analysis

Total RNA was extracted using Trizol reagent (15596026, Invitrogen) and purified up by RNeasy Micro Kit (74004, Qiagen). The integrity (18S/28S) of RNA was evaluated by agarose gel electrophoresis and the concentrations was determined using NanoDrop. The mRNA microarray profiling was performed using Affymetrix GeneChip Mouse Clariom S Array. Total RNA labelling, microarray hybridization and scanning were performed according to standard Affymetrix GeneChip Expression analysis technical protocols. Student's t‐test was employed to compare the disparities between the two groups for the identification of differentially expressed genes. These genes were regarded as differentially regulated between the two groups when the expression difference was > 1.3‐fold and the *p*‐value was < 0.05. And then, the data were analyzed with the robust multichip analysis algorithms, which included Gene Ontology (GO) enrichment analysis and functional pathway analysis using the Kyoto Encyclopedia of Gene and Genomes (KEGG). The RNA microarray data are available in the GEO database (GSE282599).

### 2.9 Cytokine/chemokine array

Protein concentrations of the skin tissue homogenates were determined by BCA assays. The skin homogenates were then used for cytokines assays as previously described.^29^ In brief, total 33 immune cytokines and chemokines were examined simultaneously with Bio‐Plex Pro™ Mouse Chemokine Assays Kit (Bio‐Rad) according to the manufacturer's protocol.

### 2.10 Western blot

Western blots were carried out in accordance with our previous study.^39^ In brief, both cutaneous and cellular protein lysates were extracted with Complete Lysis‐M buffer containing both protease inhibitors and phosphatase inhibitors (4719964001, Roche Applied Science). Protein lysates of conditioned medium (CM) were extracted with Liquid Sample Protein Purification Kit (121208, Beijing Tiandz Gene Technology Co., LTD). Protein concentrations were examined by the BCA assay kit. In each lane of the 10% SDS gel, 50 mg protein lysate was added. The protein samples were transferred onto PVDF membrane, blocked with 5% non‐fat milk and incubated with primary antibodies at 4°C overnight. The detailed information of primary antibodies used in WB is shown in Table S2B. Membranes were incubated with relevant IRDye conjugated secondary antibodies for 1 h at room temperature (1:5000, LI‐COR Biosciences). The infrared fluorescence image was obtained using Odyssey infrared imaging system (Odyssey CLx, LI‐COR Bioscience).

### 2.11 uPA activity measurement

Tissue samples of wounds were immediately frozen after collection. Then samples were homogenized and protein concentrations of wound tissue homogenates were examined by BCA assays. The uPA enzyme activity was measured using uPA activity assay (ECM600, EMD Millipore) as previously described.^29^

### 2.12 Gelatin zymography

Equal amounts of proteins from the homogenized skin tissues were subjected to zymography for the detection of MMP2 and MMP9 activities as previously reported with modifications.^30^ Briefly, samples were added to each lane and subjected to 8% sodium dodecyl sulfate(SDS)‐polyacrylamide gel electrophoresis containing 1 ×Substrate G. After electrophoresis, the gels were washed twice in 2.5% Triton X‐100 to remove SDS and further incubated at 37°Cin 0.1 M glycine‐sodium hydroxide (pH8.3) for 20 h. The gel was stained with 1% Coomassie Brilliant Blue R‐250 and destained with destaining buffer (5% acetic acid and 10% methanol) until the clear bands had been visualized. The bands of the gel enzyme spectrum were scanned and quantitatively analyzed using Image.

### 2.13 Conditioned medium (CM) preparation and M1 polarization treatment

HEK293 and RAW264.7 cells were grown in DMEM medium (Gibco) supplemented with 10% fetal bovine serum. For conditioned medium preparation, the Myc‐tagged Spink7 plasmid (MR218270, Origene, 500 or 1000 µg/well) and control vector were transfected into 293 cells in 6‐well plate using Lipofectamine 2000. Twenty‐four hours after transfection, the conditioned medium was collected, centrifuged to remove cell debris and filtered by 0.45 µm membrane, and then stored in ‐80°C refrigerator for later use. For M1 polarization treatment, RAW264.7 cells were incubated with CM of Spink7 or control for 24 h, then stimulated by lipopolysaccharide (LPS, 437627, Sigma) with concentration of 1 µg/mL for 4 h. Total RNA was extracted from the cell samples for real‐time RT‐PCR.

### 2.14 KLKs protease activity measurements

Homogenates of different skin tissues were mixed with BOC‐VPR‐AMC fluorogenic peptide substrate (99 µM, HY‐137784, MCE) and incubated for 5 min at 37°C. The plate was read by a fluorescence plate reader (SpectraMax iD3, Molecular Devices) with an excitation wavelength of 380 nm and an emission wavelength of 460 nm every minute for a total time of 1.0 h or 1.2 h.

### 2.15 Statistical analysis

Experimental data were analyzed using GraphPad Prism 6.0 software or SPSS Statistics. Data were shown as mean ± SD. Statistical analysis was performed by Student's *t* test except the data of both dynamic wound healing assays and KLKs protease activity measurements. For assessing the wound area in relation to both treatment and time, a repeated measures two‐way ANOVA was conducted. For dynamic analysis of KLKs protease activities in different time points, one‐way repeated measures ANOVA was employed. The statistical significance is indicated by asterisks as described in the figure legends (*p < 0.05, ** *p *< 0.01, *** *p *< 0.001).

## 3 RESULTS

### 3.1 Injury induced Spink7 upregulation in the differentiated epidermal granular keratinocytes of proliferative phase during skin wound healing

**FIGURE 1:**
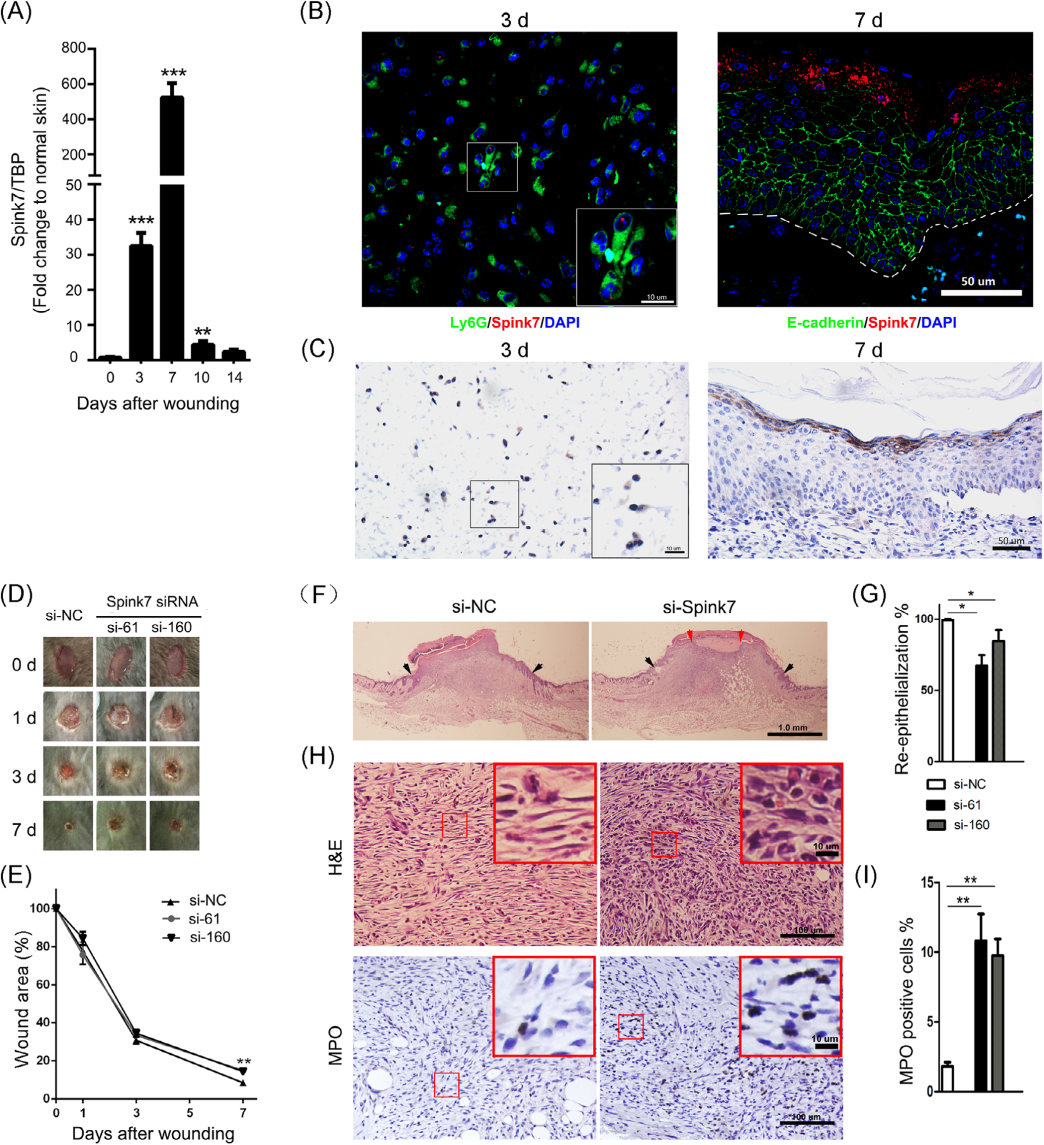
Spink7 is significantly upregulated during skin wound repair and knockdown Spink7 by siRNAs results in delayed healing. (A) Expression of Spink7 mRNA levels in skin wound healing examined by real‐time RT‐PCR (*n* = 5 per time point). (B) Double staining for Spink7 mRNA (FISH in red) and Ly6G or E‐cadherin (IF in green) to identify Spink7 mRNA transcript in wound tissues. Left panel for Day 3 wound tissue and right panel for Day 7 wound tissue. The white dashed line separates the epidermis from the dermis. (C) Representative images of IHC staining for Spink7 in Day 3 wound tissue (left panel) and Day 7 wound tissue (right panel) to show positive signals in neutrophils and keratinocytes. The local magnification image of the left panel shows neutrophils with segmented nuclei that exhibit positive signals. (D) Macroscopic appearance of wound closure in si‐NC and si‐Spink7 treated wounds in mice at Day 0, 1, 3, and 7 after wounding. (E) Quantification of residual wound area in si‐NC and si‐Spink7 treated wounds in mice. Data are expressed as the percentage of the remaining area to the initial wound area. *n* = 5 samples/group. (F) Representative H&E sections from si‐NC and si‐Spink7 treated wounds on Day 7 after wounding. Black arrows delineate wound edges. Red arrows highlight epithelial migrating tongues. (G) Quantification and calculation percentages of wound re‐epithelialization. *n* = 5 samples/group. (H) High power fields of H&E sections from si‐NC and si‐Spink7 treated day 7 wounds (up panel) and representative images of IHC staining for MPO (down panel). (I) Quantification of MPO positive neutrophils of day 7 wounds of si‐NC and si‐Spink7 treated mice (*n* = 5). Data are represented as the mean ± SD. ^∗^ *p* < 0.05, ^∗∗^ *p* < 0.01, ^∗∗∗^ *p* < 0.001. Data are representative of three independent experiments.

The IHC staining in the HPA database shows that SPINK7 exhibits strong IHC signals in suprabasal epithelium of oesophageal tissue and in epidermal stratum granulosum of skin sample (Figure S1A), which are in accordance with previous reports.^27^, ^38^ However, despite the similar expression pattern of murine Spink7 in the oesophagus to that of humans, it was not detectable in murine back skin tissue as determined by IHC (Figure S1B). Since IHC detection for Spink7 used customized antibody, we also stained the oesophageal tissue of KO mice and found almost no positive signal (Figure S1C), indicating this customized antibody has good specificity for IHC examination. To further examine the expression of Spink7 in normal murine tissues, we performed quantitative RT‐PCR analyses. Results showed that Spink7 highly expressed in oesophagus, tongue, oral mucosa and plantar skin, but not in other tissues including back skin (Figure S1D). To investigate the role of Spink7 in cutaneous wound repair, the expression patterns of Spink7 in wound closure process were examined. As shown in Figure 1A, the mRNA levels of Spink7 were significantly upregulated following skin injury, showing an initial increase on Day 3, reaching a peak on Day 7 with over 500‐fold induction compared to normal skin tissue, and returning to baseline by Day 14 post‐injury. Meanwhile, examination conducted at earlier stage of wound repair revealed that Spink7 expression was similar to that in normal skin tissue at day 1 and day 2 after wounding, and no significant increase was observed (Figure S1E). To further verify the expression pattern of Spink7 in wound repair, we performed double staining with RNA FISH for Spink7 in red fluorescence and IHC for E‐cadherin labelling epithelial cells or Ly6G labelling neutrophils in green fluorescence. The reason for choosing the double‐labelling strategy is that previous studies have reported that Spink7 is expressed in epithelial or neutrophil cells.^27^, ^29^ As Spink7 expresses constitutively in oesophageal epithelium, the validity of double staining technology was confirmed firstly in oesophageal tissue (Figure S2). Results from skin wounds indicated that Spink7 mRNAs mainly located in epidermal stratum granulosum keratinocytes in day 7 as well as Ly6G positive neutrophils in Day 3 after wounding (Figure 1B). Moreover, IHC results with customized Spink7 antibody further confirmed the expression profiles examined by FISH (Figure 1C). Weakly Spink7 IHC signals were detected in polymorphonuclear leucocytes in Day 3 after wounding (Figure 1C left panel). And intensive signals were exhibited in stratum granulosum keratinocytes in day 7 after wounding (Figure 1C right panel).

### 3.2 Topical inhibition of Spink7 by siRNA results in delayed wound healing

To explore whether the upregulation of Spink7 has an impact on skin wound healing, we attempted to downregulate endogenous Spink7 in wounds with Pluronic gel and assess its influence on wound closure. Two siRNAs targeting Spink7 were designed, and their knockdown effects were validated through co‐transfection with a Spink7‐myc‐tag plasmid in 293 cells by western blot (Figure S3A). Furthermore, these Spink7 siRNAs effectively inhibited Spink7 mRNA levels in vivo within wounds (Figure S3B). Our findings revealed that wounds treated with Spink7 siRNAs exhibited delayed healing at day 7 post‐injury compared to control wounds treated with NC siRNAs (Figure 1D,E). Subsequent morphometric analysis demonstrated that local knockdown of Spink7 significantly hindered wound re‐epithelialization (Figure 1F,G), consistent with our gross observations. Moreover, the wounds with suppressed Spink7 expression displayed more immature granulation tissue and a higher presence of infiltrated neutrophils compared to control wounds (Figure 1H,I).

### 3.3 Spink7 KO mice show impaired wound closure with sustaining neutrophil infiltration

**FIGURE 2:**
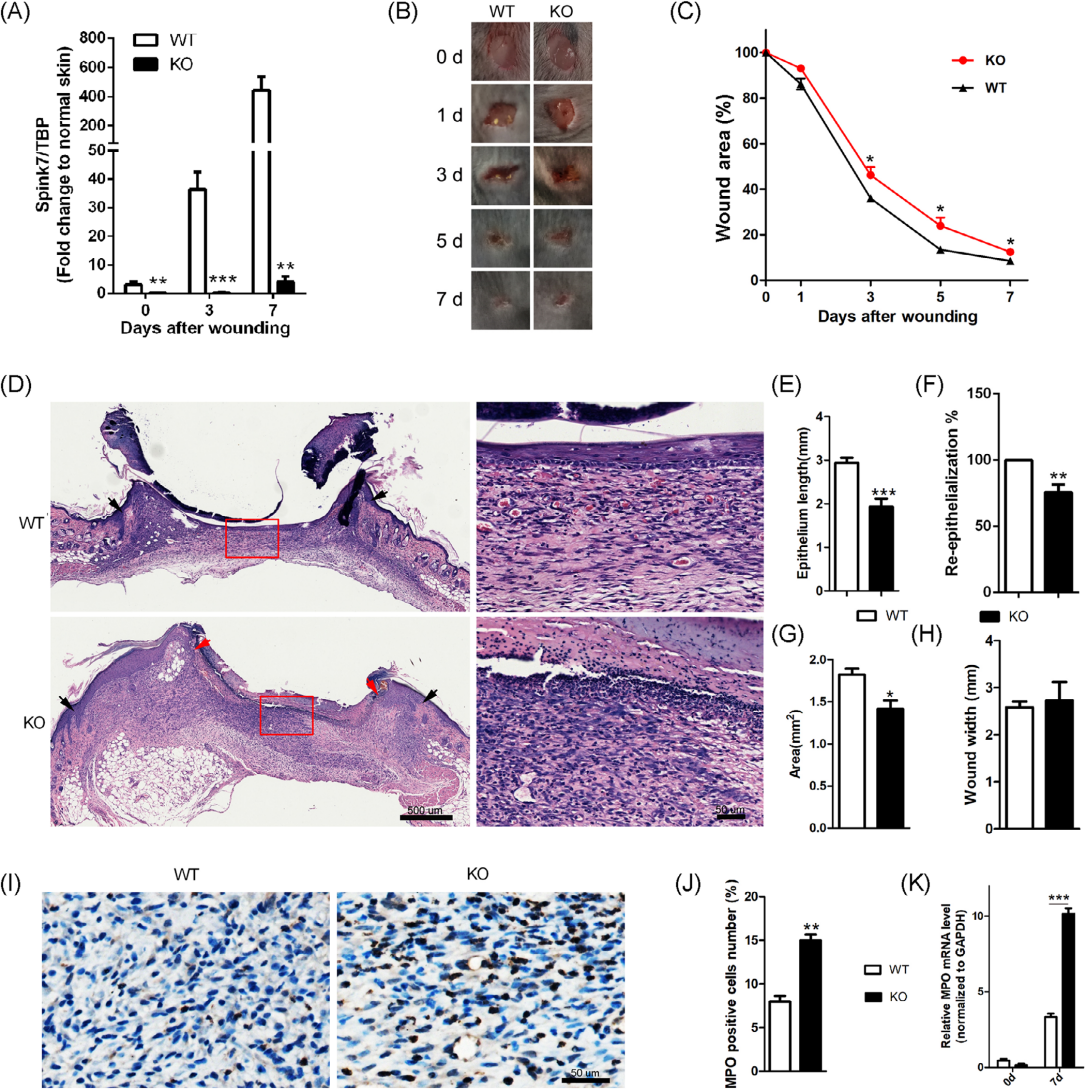
Spink7 KO mice show impaired wound closure. (A) Expression of Spink7 in both KO and WT mice during wound healing examined by real‐time RT‐PCR (n = 5 per time point). (B) Macroscopic appearance of wound closure in wounds of KO and WT mice at different time points. (C) Quantification of wound area at different time points in wounds of KO and WT mice. *n *= 5 samples/group. (D) Representative H&E sections in 7‐day wounds of KO and WT mice. Black arrows delineate wound edges. Red arrows highlight epithelial migrating tongues. (E) Quantification of the epithelial tongue lengths of 7‐day wounds. *n* = 6–8 samples/group. (F) Quantification and calculation percentages of wound re‐epithelialization. *n *= 6–8 samples/group. (G) Quantification of the wound areas of 7‐day wounds. *n* = 6–8 samples/group. (H) Quantification of the wound widths of 7‐day wounds. *n* = 6–8 samples/group. (I) Representative images of IHC staining for MPO in 7‐day wounds of KO and WT mice. (J) Quantification of MPO positive neutrophils. *n* = 6–8 samples/group. (K) Expression of MPO mRNA levels in 7‐day wounds of KO and WT mice examined by real‐time RT‐PCR. n = 6–8 samples/group. Data are represented as the mean ± SD. ^∗^ *p* < 0.05, ^∗∗^ *p* < 0.01, ^∗∗∗^ *p* < 0.001 versus WT mice group at same time points. Data are representative of three independent experiments.

Given that Spink7 expression is significantly upregulated during the proliferative phase of wound repair, and siRNAs knockdown of Spink7 leads to delayed healing characterized by excessive inflammatory response, we further confirmed the role of Spink7 in skin wound repair using KO mice. The mRNA levels of Spink7 in wounds at various stages of healing were almost completely absent in Spink7 KO mice (Figure 2A). In accordance with the outcomes of local silencing of Spink7 in skin repair, the wounds of Spink7 KO mice exhibited significantly impaired healing from Day 3 to Day 7 post‐injury (Figure 2B,C). Histologically, the proliferative phase wounds (Day 7 post‐injury) of Spink7 KO showed increased infiltration of inflammatory cells as observed under a microscope (Figure 2D). And morphometric analyses revealed that the wounds of KO mice had reduced epithelial migrating length, delayed re‐epithelization and smaller wound areas (Figure 2E–G), despite no significant impact on wound widths (Figure 2H). Furthermore, in comparison with WT mice, the infiltration of MPO‐positive neutrophils and the expression level of MPO mRNA in the wounds of Spink7 KO mice on Day 7 were significantly increased (Figure 2I–K).

### 3.4 Loss of Spink7 induces excessive proinflammatory cytokines and chemokines

**FIGURE 3:**
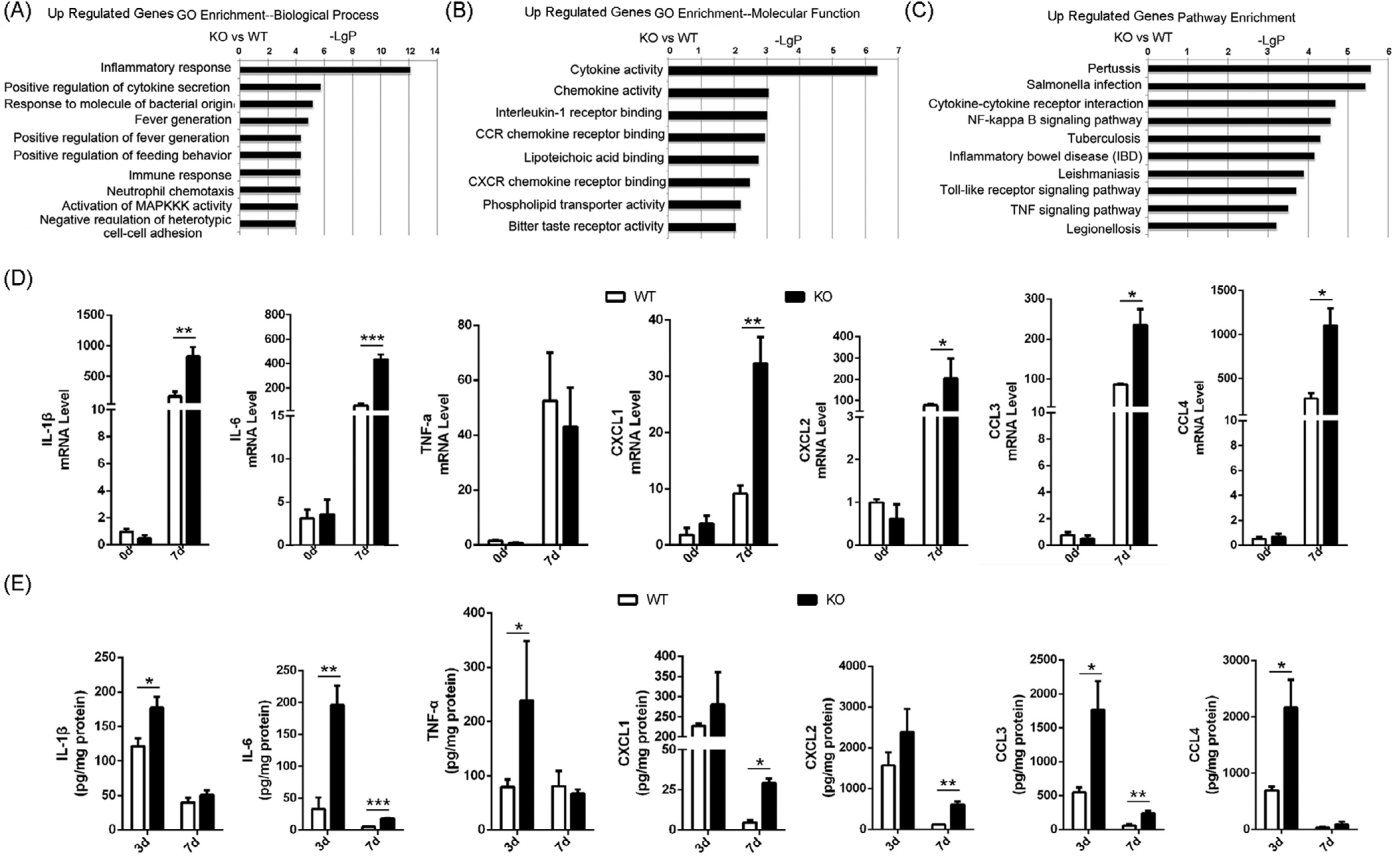
Loss of Spink7 augments inflammatory response and enhances production of chemokines/cytokines during wound healing. (A) Graph of top 10 biological process terms for GO analysis from up‐regulated genes of microarray experiments of 7‐day wound samples (KO vs WT). (B) Top 10 molecular function terms for GO analysis. (C) Top 10 pathway terms for KEGG analysis from up‐regulated genes of microarray experiments. (D) Examining some proinflammatory chemokines/cytokines in wound tissue by real‐time RT‐PCR for validation of partial results of microarray experiments. *n* = 5 samples/group. Representative data are shown from two independent experiments. (E) Protein levels of some proinflammatory chemokines/cytokines in wound tissue detected by Bio‐Plex Pro™ Mouse Chemokine Assays Kit. *n* = 4–6 samples/group. Representative data are presented as the concentrations of chemokines/cytokines in total protein from two independent experiments performed in duplicate. Data are represented as the mean ± SD. * *p *< 0.05, ** *p *< 0.01 and *** *p *< 0.001 versus WT mice group at same time points.

To investigate the potential mechanism underlying the delayed wound healing caused by Spink7 loss, microarray experiments were performed on 7‐day wound tissues of WT and KO mice (Figure S4A). We found 491 genes significantly differentially expressed in Day 7 wounds between WT and KO mice (*p *< 0.05, fold change > 1.3), and focused on the 282 upregulated differentially genes which had higher values of ‐LgP in analyses of both up regulated genes GO and pathway enrichments than those of down regulated genes. As seen in Figure 3A, the topmost significant up regulated genes enrichment GO were biological processes related to inflammatory response, positive regulation of cytokine secretion and response to molecule of bacterial origin. Consistently, the topmost GO in molecular function were cytokine activity, chemokine activity and interleukin‐1 (IL‐1) receptor binding (Figure 3B). And the topmost up regulated genes KEGG pathway enriched in pertussis, salmonella infection and cytokine‐cytokine receptor interaction (Figure 3C). The results of downregulated genes enrichment GO in biological process and molecular function as well as KEGG pathway are also provided in Figure S4B–D. Furthermore, the results of microarray experiments were partially verified by examining some important proinflammatory cytokine/chemokine genes by qRT‐PCR. The mRNA levels of cytokine/chemokine such as IL‐1β, IL‐6, CXCL1/2, and CCL3/4 were all significantly higher in wounds of KO mice than those of WT mice, except for TNF‐α (Figure 3D). Further investigation showed protein levels of these cytokines and chemokines were significantly up‐regulated in the wounds of KO mice on day 3 and/or 7 compared with those of WT mice, which were detected with wounds homogenates by a multiplex cytokine/chemokine array (Figure 3E).

### 3.5 Loss of Spink7 results in impaired M2 polarization of macrophages in wounds

**FIGURE 4:**
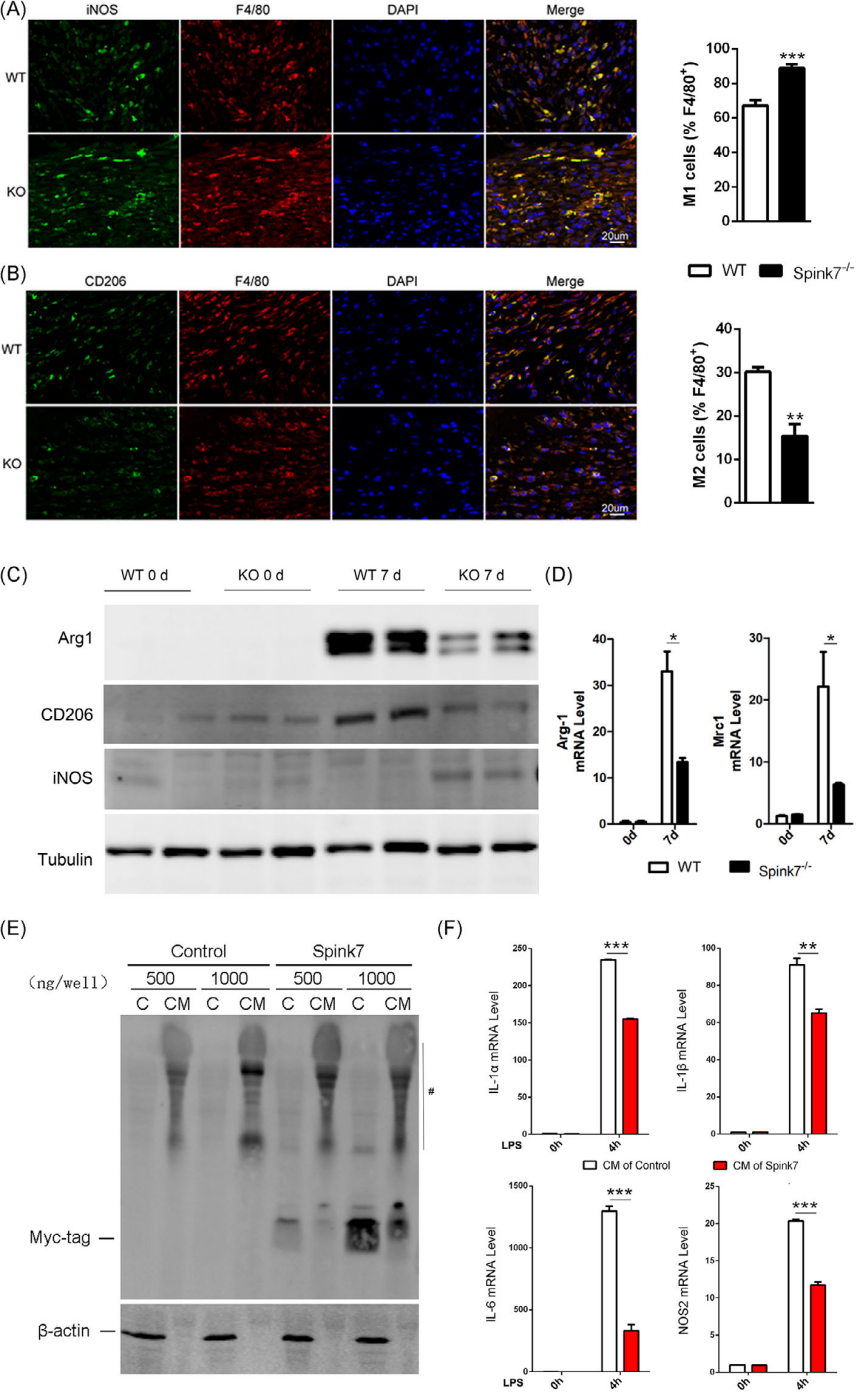
Loss of Spink7 results in impaired M2 polarization of macrophages in wounds. (A) Double staining for F4/80 and iNOS to identify M1 macrophages in 7‐day wound tissues. Left panel for representative images and right panel for quantification analysis. *n* = 6 samples/group. (B) Double staining for F4/80 and CD206 to identify M2 macrophages in 7‐day wound tissues. Left panel for representative images and right panel for quantification analysis. *n* = 6 samples/group. (C) Examining protein levels of Arg1, CD206 and iNOS in 7‐day wound tissues of KO and WT mice. The experiments were performed in triplicate. (D) Detecting mRNA levels of Arg1 and Mrc1 in 7‐day wound tissues of KO and WT mice. *n* = 5 samples/group. (E) Secreted Spink7 protein was examined in conditioned medium. Total proteins were extracted from conditioned media (CM) and cells (C) for WB using Myc‐tag antibody. #, the non‐specific bands in samples from CM. (F) Levels of IL‐1α, IL‐1β, IL‐6 and iNOS mRNA were evaluated by RT‐PCR in CM‐treated LPS‐induced RAW264.7 M1 polarization samples. Data are represented as the mean ± SD. *p < 0.05, ** *p *< 0.01 and *** *p *< 0.001. Data are representative of three independent experiments.

Macrophage polarization plays an important role in the resolution of inflammation during skin wound healing.^6^, ^7^, ^8^ As loss of Spink7 led to impaired wound closure characterized by excessive inflammatory response, we asked whether disruption of M2 polarization involved in these phenotypes of Spink7 KO mice. Then, we labelled the F4/80 positive macrophages with either M1‐specific iNOS antibody or M2‐specific CD206 antibody in proliferative phase (day 7 wounds) as previously described.^43^ As seen in Figure 4A,B, loss of Spink7 increased the number of iNOS positive M1 macrophages and decreased the number of CD206 positive M2 macrophages in the wounds of Spink7 KO mice compared to those of WT mice. Subsequently, western blot analyses were conducted, revealing that the protein levels of the M2 marker Arg1 and CD206 decreased, while the M1 marker iNOS increased in wound samples from knockout (KO) mice. (Figure 4C), which were in good concordance with the observation of double immunofluorescent labelling experiments. Furthermore, mRNA levels of both Arg1 and Mrc1 (the gene encoding for CD206) were examined with qRT‐PCR and the results showed significantly downregulated of these two M2 marker genes in wounds samples from KO mice (Figure 4D). As macrophages exhibited minimal expression of Spink7 protein and mRNA, we speculated that secreted Spink7 may influence the polarization of macrophages through paracrine signalling. And indeed, secreted Spink7 could be detected in the conditioned medium (CM) 24 h after transfection of the Spink7‐myc‐tag plasmid into HEK293 cells (Figure 4E). Then, the effects of CM on LPS‐induced M1 polarization of macrophages were investigated by examining proinflammatory genes with RT‐PCR. The results showed that CM containing Spink7 treatment could significantly reduce the mRNA levels of Il‐1α, Il‐1β, Il‐6 and Nos2 (Figure 4F).

### 3.6 Loss of Spink7 leads to increased uPA and MMP2/9 activities in wounds

**FIGURE 5:**
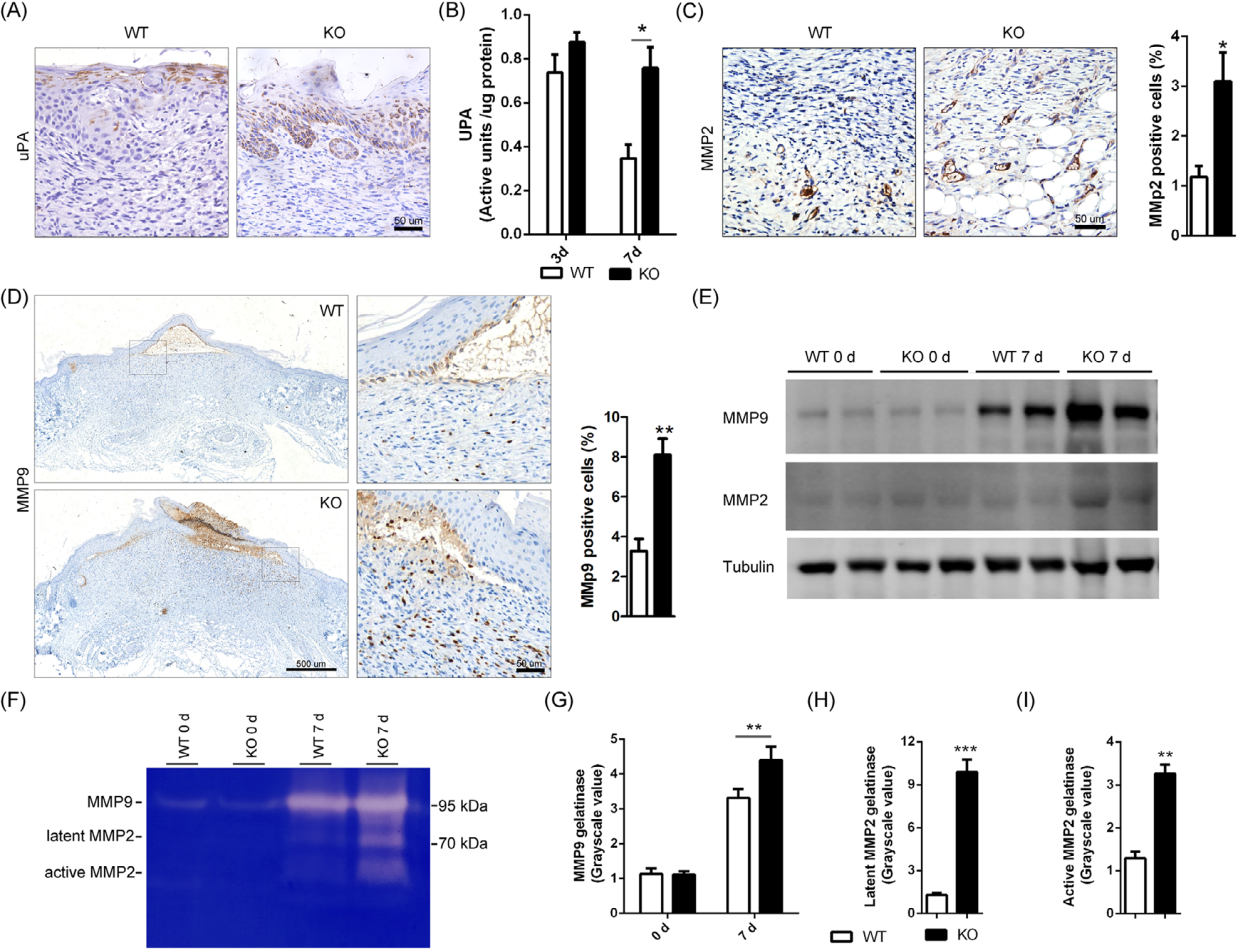
Loss of Spink7 leads to increased uPA and MMP2/9 activities in wounds. (A) Representative images of IHC staining for uPA in 7‐day wounds of KO and WT mice. (B) Analysis of uPA activity of both 3‐day and 7‐day wounds. *n* = 5 samples/group. (C and D) IHC staining forMMP2 and MMP9 in 7‐day wounds of KO and WT mice. Left panel for representative images and right panel for quantification analysis. (**E**) Detection of MMP9 and MMP2 protein levels in 7‐day wounds between KO and WT mice. The images are representative of experimental triplicates. (F) Gelatin zymography for MMP2/9 activities of 7‐day wounds. The images are representative of experimental triplicates. (G–I) Quantitative analysis of grayscale scanning of bands in gel zymography for MMP9, latent MMP2 and active MMP2. Data are represented as the mean ± SD. *p < 0.05, ** *p *< 0.01 and *** *p *< 0.001. Data are representative of three independent experiments.

It has been reported that human SPINK7 serves as serine protease inhibitor suppressing proteolysis activities of uPA and MMP2.^30^, ^31^, ^32^ As both uPA and MMPs play critical roles in regulating inflammatory response,^17^, ^18^, ^19^, ^44^, ^45^ we speculated that the overactivated proteolysis activities may be involved in delayed wound healing phenotype of Spink7 KO mice. Then, the expression of uPA in wounds from both WT and KO mice of day 7 after wounding were initially examined by IHC. The expression of uPA is mainly localized to the newly formed epidermal cells near the repair area, but there is a difference in the distribution of positive signals between the two groups. As seen in Figure 5A, there were dispersed positive uPA signals in cells of granular layer in WT mice samples, but continuous and extensive positive signals in almost full thickness of wound epidermis in KO mice samples. And indeed, the results of uPA activity assessment from wound homogenates indicated that 7‐day wound tissues in KO mice had significantly higher uPA activities than those of WT controls, which showed similar levels as the 3‐day wounds (Figure 5B). Furthermore, IHC for both MMP2 and MMP9 showed that there were more intensive positive signals in KO mice wound sections than those of WT controls, which were further confirmed by semi‐quantitative analysis results (Figure 5C,D). However, there are differences in the expression patterns between MMP2 and MMP9. MMP2 is mainly distributed in deep granulation tissue, with a marked staining of the microvascular wall (Figure 5C). In contrast, MMP9 is mainly distributed in the newly formed epidermal basal keratinocytes and infiltrating inflammatory cells near the repair area (Figure 5D). Additionally, we utilized WB to examine the protein levels of MMP2/9 and discovered higher protein levels of these two MMPs in KO wound samples than those of WT controls (Figure 5E). What's more, conventional gelatin zymography was performed to assess whether Spink7 affects enzymatic activities of both MMP2 and MMP9 (Figure 5F). Upon conducting grayscale scanning and quantitative analysis of the bands in the gel zymography, it was found that the enzyme activity of MMP9 in the 7‐day wound of KO mice exceeded that of WT mice (Figure 5G). Meanwhile, the enzymatic activities of both latent and active MMP2 exhibited significantly increased in 7‐day wound samples of Spink7 KO mice (Figure 5H,I).

### 3.7 Loss of Spink7 exhibits enhanced KLKs activities, but inhibiting PAR2 signalling exacerbates the phenotype of impaired wound closure

**FIGURE 6:**
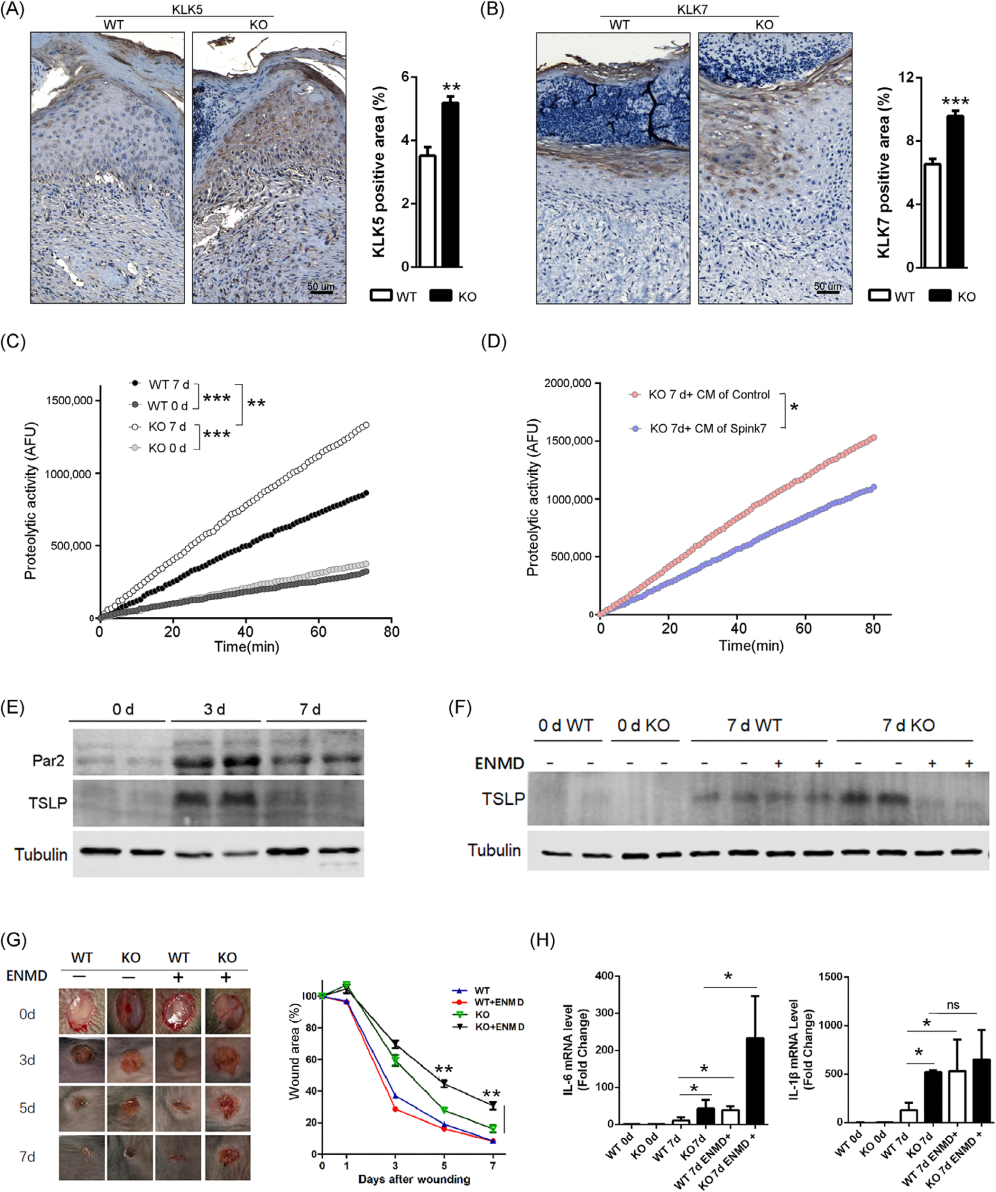
Loss of Spink7 exhibits enhanced KLKs activities, but inhibiting PAR2 signalling exacerbates the phenotype of impaired wound closure. (A) Representative images of IHC staining for KLK5 in 7‐day wounds of KO and WT mice. Right panel for quantification analysis. *n* = 5 samples/group. (B) Representative images of IHC staining for KLK7 in 7‐day wounds of KO and WT mice. Right panel for quantification analysis. *n* = 5 samples/group. (C) Trypsin‐like proteolytic activity measurement of normal skin and 7‐day wound tissues from KO and WT mice. *n* = 4 samples/group. (D) Evaluation of ability to inhibit proteolytic activity of KO wound samples by addition of CM containing Spink7 protein. The experiments were performed in triplicate. (E) Detection of PAR2 and TSLP protein levels in wounds of WT mice at different time points by WB. (F) Examination of TSLP protein levels in wounds to determine the effect of selective inhibition of PAR2 by ENMD‐1068 during wound healing. (G) Macroscopic appearance and quantification analysis of wound closure in both WT and KO mice treated with ENMD‐1068. Right panel for quantification analysis. *n* = 5 samples/group. (H) Examining mRNA levels of IL‐6, and IL‐1β in wounds treated with ENMD‐1068 by real‐time RT‐PCR. *n* = 5 samples/group. *p < 0.05, ** *p *< 0.01 and *** *p *< 0.001. Data are representative of two to three independent experiments.

Previous research on EoE suggested that SPINK7 inhibits the inflammatory response by suppressing KLK5 as well as the downstream protease‐activated receptor 2 (PAR2) signalling pathway.^28^ KLK5 is the primary activator of the cascade of KLKs, which play crucial roles in regulating skin desquamation and inflammation.^46^, ^47^ Additionally, studies have shown that KLK5 enhances inflammatory response through both PAR2 dependent and independent mechanisms in bronchial epithelium.^48^, ^49^ Therefore, we investigated the role of interaction between Spink7 and KLKs in delayed wound healing of Spink7 KO mice. Initially, immunohistochemical staining was employed to examine the expression of KLK5 as well as its downstream KLK7 in normal murine skin tissues. The results showed that both KLK5 and KLK7 were predominantly expressed in the stratum granulosum of normal epidermis from both WT and KO mice (Figure 5), consistent with their roles in desquamation. Following injury, loss of Spink7 led to upregulated KLK5 expression in 7‐day wound samples (Figure 6A). Meanwhile, there were significantly increased positive signals of KLK7 in KO mice wound sections compared to those of WT mice (Figure 6B). Furthermore, analysis of trypsin‐like serine proteases activity in skin and wound lysates revealed an elevated proteolytic activity in wound samples from Spink7 KO mice compared with those from WT control mice (Figure 6C). Moreover, the bioactive Spink7 secreted in the conditioned media (CM) inhibited the proteolytic activity of recombinant mouse KLK5 (Figure S6). Notably, CM containing secreted Spink7 significantly suppressed activity of trypsin‐like serine proteases in wound samples from KO mice (Figure 6D). Subsequently, we asked whether inhibiting the KLKs‐PAR2 signalling could rescue the delayed wound healing phenotype observed in KO mice. As shown in Figure 6E, PAR2 signalling turned out to be activated during normal wound healing as evidenced by Western blot analysis for PAR2 and downstream effector TSLP. We then examined the role of PAR2 activation in wound healing using a selective antagonist ENMD‐1068 in vivo for both WT and KO mice. Treatment with ENMD‐1068 suppressed TSLP production induced by loss of Spink7 at day 7 post‐wounding (Figure 6F). Unexpectedly, inhibiting PAR2 activation exacerbated impaired wound healing in Spink7 KO mice (Figure 6G). In accordance with these observations, ENMD‐1068 treatment resulted in enhanced expression levels of IL‐6 and IL‐1β detected by RT‐PCR analysis (Figure 6H).

### 3.8 Inhibition of Spink7 promotes wound healing in R‐W‐CI model by augmenting inflammation

One significant characteristic of wounds in RCI is delayed healing due to impaired inflammatory response, which aggravates the overall injuries and significantly increases the risk of infection in the early stage.^10^, ^11^, ^12^, ^13^ As previously described, wounds from R‐W‐CI mice showed lack of inflammation in Day 3 and reduced granulation as well as re‐epithelialization on Day 7 compared to those of wounded‐only mice (Figure S7A,B). Microarray experiments were then employed to explore the potential mechanisms of delayed wound healing in R‐W‐CI using samples from Day 3 wounds. Consistent with the pathological feature of impaired inflammation, the topmost significant downregulated genes enrichment GOs (R‐W‐CI vs. W) were biological processes related to immune system process, integrin‐mediated signalling pathway, inflammatory response, innate immune response, and so on (Figure S7C, right panel). And the topmost up regulated genes enrichment GOs were biological processes mainly related to metabolic processes, notably negative regulation of peptidase activity (Figure S7C, left panel). Similar variation tends were also observed in KEGG pathway analyses, in which inflammatory response signalling pathways were downregulated and pathway of peptidases as well as inhibitors was significantly upregulated (Figure S7D). These results suggested that delayed healing wounds of RCI had reduced inflammation accompanied with dysregulation of protease inhibitors, which suggested that manipulating protease activities may improve wound healing of R‐W‐CI.

**FIGURE 7:**
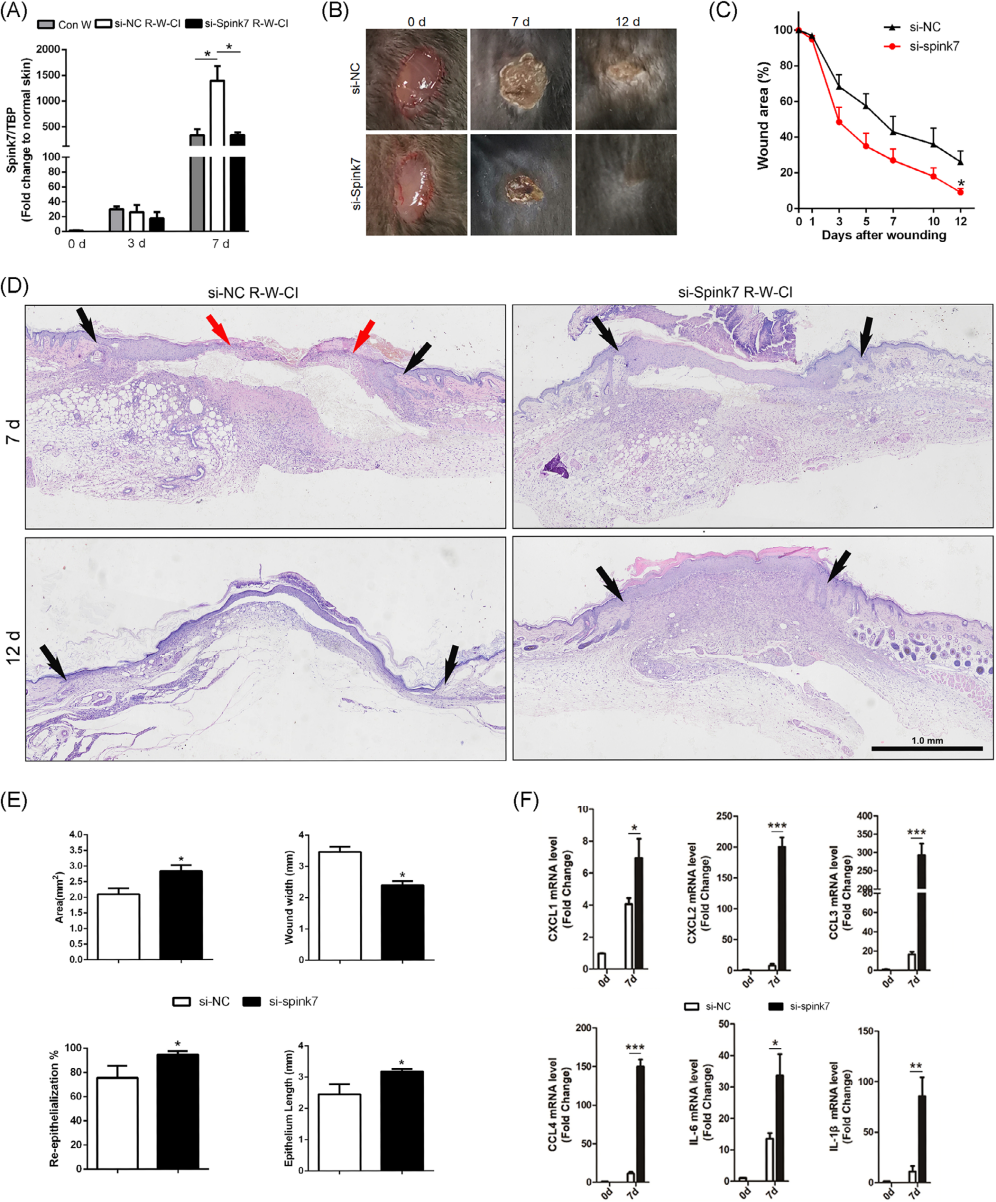
Knockdown of Spink7 promotes wound healing in R‐W‐CI model by augmenting inflammation. (A) Expression of Spink7 mRNA levels in wounds of R‐W‐CI and the in vivo knockdown effects of siRNAs gel against Spink7 examined by real‐time RT‐PCR (n = 5 per time point). (B) Macroscopic appearance of wound closure in si‐NC and si‐Spink7 treated wounds in R‐W‐CI mice at day 0, 7, and 12 after wounding. (**C**) Quantification of wound area in si‐NC and si‐Spink7 treated wounds in R‐W‐CI mice. *n *= 5 samples/group. (D) Representative H&E sections from si‐NC and si‐Spink7 treated wounds in R‐W‐CI mice on day 7 and 12 after wounding. Black arrows delineate wound edges. Red arrows highlight epithelial migrating tongues. (E) Quantification of wound areas, wound widths, percentages of re‐epithelialization and epithelial tongue lengths from si‐NC and si‐Spink7 treated wounds in R‐W‐CI mice on Day 7 after wounding. n = 5 samples/group. (F) Examining mRNA levels of CXCL1/2, CCL3/4, IL‐6, and IL‐1β in si‐NC and si‐Spink7 treated 7‐day R‐W‐CI wounds by real‐time RT‐PCR. *n* = 5 samples/group. *p < 0.05, ** *p *< 0.01 and *** *p *< 0.001. Data are representative of three independent experiments.

Then, we asked whether the serine protease inhibitor Spink7 knockdown could promote healing of wounds in R‐W‐CI through augmenting inflammation. As depicted in Figure 7A, samples from Day 7 wounds of R‐W‐CI mice showed significantly higher Spink7 mRNA levels than those of wounded‐only mice, and the treatment with siRNAs gel against Spink7 could reduce mRNA levels in R‐W‐CI wounds to a level similar to that of normal wounds. Further IHC detection revealed that the expression of Spink7 was mainly located in the newly thickened epidermis in Day 7 wounds of R‐W‐CI, but the positive signals were more extensive than that of wounded‐only mice (Figure S8 top panel). Meanwhile, expression of Spink7 was significantly attenuated in the wounds treated with si‐Spink7 gel (Figure S8 bottom panel). Consequently, the wounds treated with si‐Spink7 in the R‐W‐CI model healed more rapidly compared to the control wounds treated with si‐NC (Figure 7B,C). Moreover, the histopathologic characteristics of H&E sections indicated that Spink7 knockdown improved the impaired wound healing of R‐W‐CI model by promoting re‐epithelialization and alleviating dysplasia of granulation tissue (Figure 7D). Further morphometric analyses confirmed both macroscopic and histopathologic observations. Local knockdown of Spink7 promoted wound re‐epithelialization with longer epithelial tongue and enhanced growth of granulation tissue with larger wound areas and smaller wound widths (Figure 7E). What's more, knockdown of Spink7 resulted in upregulated mRNA levels of proinflammatory chemokines and cytokines including CXCL1/2, CCL3/4, IL‐6, and IL‐1β in day 7 wounds of R‐W‐CI mode (Figure 7F), which indicated that downregulation of Spink7 could augment inflammation and then promote repair in wounds of RCI.

## 4 DISCUSSION

**FIGURE 8:**
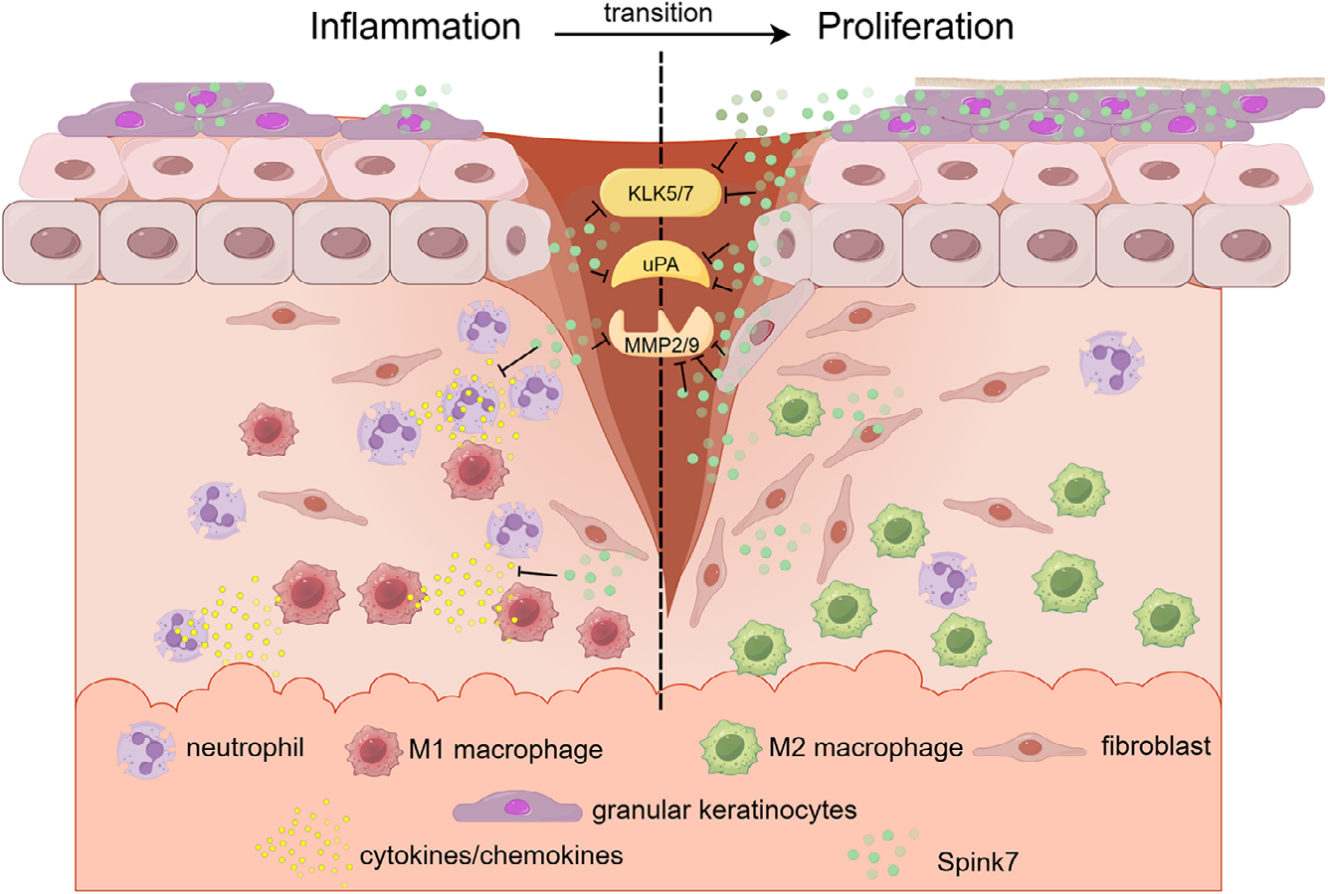
Schematic diagram showing the mechanism of promoting inflammation resolution by Spink7 during skin wound healing.

Recent reports have indicated that SPINK7 is a serine protease inhibitor having ability to inhibit uPA and MMP2 activities, which plays important roles in tumorigenesis and development of oesophagus cancer.^30^, ^31^, ^32^, ^33^, ^34^ Previous studies identify SPINK7 as an inhibitory checkpoint for oesophageal epithelial inflammatory responses, which plays important role during development of eosinophilic oesophagitis.^27^, ^28^ Our previous work shows that Spink7 exerts an important protective role in experimental colitis.^29^ In this report, we have demonstrated that the antiprotease Spink7, which originates from epidermal granular keratinocytes of proliferative phase, plays a key role in skin wound healing by promoting the resolution of inflammation through the regulation of multiple proteases activities (Figure 8), which presents one promising target for the prevention and management of wound healing disorders caused by dysregulated inflammatory responses.

The expression pattern of a specific gene is typically intricately related to its functionality. SPINK7 is consistently expressed at high levels in the human oesophageal epithelium, and studies have demonstrated its substantial contribution to the pathogenesis of oesophageal cancer and eosinophilic oesophagitis.^27^, ^33^, ^50^ In silkworm (Bombyx mori), SPINK7 is exclusively expressed in haemocytes, and research has shown its important role in immune defence against fungal infections.^51^ Our previous study revealed that Spink7 derived from neutrophils exerts a protective role by controlling chemokines/cytokines production in experimental murine colitis.^29^ In this work, we reported that Spink7 upregulates significantly and reaches the highest level in day 7 during murine cutaneous wound healing process. Further findings from both FISH and IHC revealed that Spink7 is predominantly expressed in stratum granulosum of hyperplastic epidermis during the proliferative phase. Moreover, our study demonstrated that the skin injury‐induced Spink7 plays a key role in promoting the resolution of inflammation. Recent research indicates that differentiation status of epidermal keratinocytes dictates their immune response, and differentiated keratinocytes dampen inflammation through ZNF750‐mediated inhibition of pattern recognition receptors.^15^ Our results suggest that similar novel biological model might exist during wound healing, where differentiated keratinocytes in the thickened epidermis of proliferative phase promote the transition from inflammation to proliferation by upregulating Spink7 (Figure 8).

Results of functional studies from both knockdown and knockout of Spink7 have mutually confirmed that deficiency of Spink7 resulted in impaired wound closure, characterized by an increased presence of residual infiltrated neutrophils during the proliferative phase (7 day after wounding). Further analysis from both microarray experiments and multiple ELISA assays revealed that loss of Spink7 enhanced inflammation response signalling pathways and promoted productions of proinflammatory chemokines/cytokines, consistent with previous reports in both murine colitis and human oesophageal epithelium models.^27^, ^29^ These suggest that the upregulation of Spink7 in the proliferative phase may exert key influence on resolving inflammation. Given the importance of neutrophils, macrophages, and cytokines in proper inflammation resolution, excessive proinflammatory cytokines may prolong neutrophil survival and disrupt macrophage polarizaiton.^6^, ^7^, ^8^, ^52^, ^53^ And indeed, loss of Spink7 results in impaired M2 polarization of macrophages in wounds of proliferative phase. What's more, conditioned medium containing Spink7 protein was able to inhibit LPS‐induced M1 polarization, which suggested that Spink7 may promote resolution of inflammation through modulating macrophage polarization via paracrine action. As for the mechanism by which Spink7 regulates macrophage polarization, we will explore it in our subsequent research. We believe that we can approach it from two perspectives. On one hand, Spink7 may inhibit the activity of many proteases, such as uPA and MMPs, thereby changing the microenvironment of macrophages and promoting M2 polarization. On the other hand, it has been reported that some members of the SPINKs family, such as SPINK9 and SPINK4, can function by activating cell receptors such as EGFR,^25^, ^54^ so we speculate that Spink7 may regulate macrophage polarization through specific receptor signalling pathway. In any case, we believe that the mechanism behind this interesting phenomenon is worth further attention.

Tight regulation of both proteases and their inhibitors is important for successful wound healing as well as proper inflammation resolution.^1^, ^17^, ^18^, ^19^ Up to skin injury, proteolytic‐induced extracellular matrix (ECM) degradation and reshaping is a key aspect of tissue repair, helping leucocytes infiltration, re‐epithelialization and angiogenesis. While controlled regulation of proteases activities is one critical component of proper wound healing, sustained high levels of proteases activities can disrupt the equilibrium between tissue damage and repair, leading to excessive ECM degradation and persistent inflammation associated with impaired wound healing. Our findings have demonstrated that the absence of Spink7 leads to increased proteolytic activities of uPA, MMP2/9, and KLKs. As uncontrolled proteases activities are common features of chronic non‐healing wounds, we believe that the persistent overactivation of the proteolytic microenvironment is the principal cause of impaired wound closure in Spink7 deficiency. In fact, uPA, uPAR, and/or their interaction are important for adhesion, migration and recruitment of leucocytes, thereby influencing the process and outcome of injuries or diseases.^55^, ^56^, ^57^ Previous study has demonstrated that downregulated SPINK7 leads enhanced uPA activity in EoE, which directly activates eosinophils by excessive uPAR cleavage.^27^ In addition, uPA can effectively inhibit efferocytosis of neutrophils by macrophages,^58^ which is key step for M2 polarization and inflammation resolution. As for MMP2/9, they play pivotal role in controlled degradation of the ECM during normal wound healing, which helps cell migration and granulation tissue remodelling.^17^, ^18^, ^19^ Elevation in levels of MMP2/9 in fluids were common features of chronic non‐healing wounds, leading an imbalance in the deposition and degradation of ECM and a state of chronicity.^20^, ^21^, ^22^, ^23^ We also demonstrated that deficiency of Spink7 leads to increased activities of KLKs including KLK5 and KLK7, which are important for epidermal desquamation process. Although the roles of KLK5 and KLK7 in inflammatory dermatoses like Netherton syndrome and atopic dermatitis have been revealed,^59^, ^60^, ^61^ their functions in wound healing are still unclear. As absence of SPINK7 leads to a marked activation of theKLK5‐PAR2 signalling pathway and promotes enhanced cytokines production in EOE model,^28^ we tried to explore the role of PAR2 activation in phenotype of impaired wound closure of Spink7 KO mice. However, inhibition of PAR2 activation further aggravated the phenotype of impaired wound healing KO mice. This result is similar to the data presented in Spink5/PAR2 double KO mice, which does not rescue the inflammatory phenotype of Spink5 KO mice.^62^ Previous reports indicated that PAR2 activation facilitated proper wound healing in mice,^63^, ^64^ which suggested that KLKs‐PAR2 activation in Spink7 KO mice may be not the main reason of impaired wound closure phenotype. Studies also showed that KLK5 enhanced cytokines secretion through both PAR2 dependent and independent mechanisms in bronchial epithelium.^48^, ^49^ Therefore, we suppose that increased KLKs activities may lead to augmented inflammatory response through PAR2 independent signals during wound healing of Spink7 KO mice.

Considering the significant role of Spink7 in modulating inflammation during wound healing, it is plausible to speculate that Spink7 may exert a crucial regulatory function in injuries associated with inflammatory dysregulation. Impaired inflammatory response is one important feature of wounds in RCI, which results in delayed healing.^10^, ^11^, ^12^ Previous study showed upregulated peptidase inhibitory activity in GO analysis in irradiated skin tissues,^65^ which is consistent with our results of microarray experiments from wound samples of RCI. Indeed, significantly induced expression of Spink7 was observed in wounds of R‐W‐CI mice. Previous studies have shown that DNA damage can upregulate SPINK7 expression at the transcriptional level through activation of the p53 pathway, subsequently participating in the regulation of the damage response.^35^, ^36^, ^37^ Whether the DNA damage‐induced p53 pathway contributes to the further upregulation of Spink7 in wounds of R‐W‐CI deserves follow‐up research. Our investigation reveals that simple downregulation of Spink7 alone is sufficient to significantly enhance the inflammatory response and promote wound healing in RCI. Generally, it is believed that the insufficient inflammatory response in wounds of RCI is primarily attributed to ionizing radiation‐induced damage of the bone marrow hematopoietic system.^10^, ^11^, ^12^ For instance, total body irradiation of 6 Gy in this model is adequate to cause severe injury of the hematopoietic system, resulting in a significant reduction in white blood cell count of peripheral blood. Our study has demonstrated that the presence of local inflammatory response suppressor in wounds of RCI, such as upregulated Spink7. In the field of RCI research, stem cell transplantation is generally regarded as an ideal strategy to promote wound healing.^10^, ^11^, ^12^ Nevertheless, constrained by ethical considerations and potential tumour risks, its implementation in practical applications is often challenging. Our preliminary study showed that Spink7 as the therapeutic target to regulate the inflammatory response of the RCI wounds can also achieve good healing effect. This suggests that modulating the inflammatory response of the wound might be an alternative strategy to promote the healing of RCI.

Taken together, our data indicate that wound‐induced expression of Spink7 promotes inflammation resolution by suppressing multiple proteases activities during wound healing. Our work delineates a novel mechanism at biological level, where the differentiated granular keratinocytes of the proliferative phase inhibit the activities of multiple proteases in the wound microenvironment through a paracrine fashion by secreting Spink7. As a checkpoint molecule for inflammatory responses, Spink7 enables the transition of wound repair from the inflammation to proliferation (Figure 8). At the medical level, it suggests a possible novel therapeutic target for impaired wounds caused by inflammatory dysregulation.

## AUTHOR CONTRIBUTIONS

Tao Wang, Yongping Su, and Na Zhao designed the experiments. Na Zhao, Guojian Wang, Shuang Long, and Xiaofan Lv performed the experiments. Tao Wang, Na Zhao, Guojian Wang, Xinze Ran, Junping Wang analyzed the data, Tao Wang and Na Zhao wrote the manuscript. All the authors reviewed and approved the final version of the manuscript.

## CONFLICT OF INTEREST STATEMENT

The authors declare no conflict of interests.

### ETHICS STATEMENT

All animal experiments were approved by Laboratory Animal Welfare and Ethics Committee of the Army Medical University (Permit Number: 82172219). All surgery was performed under pentobarbital sodium anaesthesia with efforts to minimise animal suffering.

## Supporting information



Supporting Information

Supporting Information

## Data Availability

Microarrays data have been deposited in the NCBI GEO database under accession codes GSE282599 (https://www.ncbi.nlm.nih.gov/geo/query/acc.cgi?acc=GSE282599). All the other data generated, analyzed, or processed in this study are available upon reasonable request from the corresponding author.
