# Supplementary material for: The antiprotease Spink7 promotes inflammation resolution by modulating multiple proteases activities during wound healing
Source: Clin Transl Med. 2025 Mar 27;15(4):e70291. doi: 10.1002/ctm2.70291 (PMC11949503; doi:10.1002/ctm2.70291)

**Supplementary figure legends**

**Supplementary figure 1. Expression patterns of SPINK7/Spink7 in esophagus and skin tissues from human and mouse.** (**A**) Representative images of IHC staining for SPINK7 in human esophagus (left panel) and skin (right panel) tissues. Image credit: Human Protein Atlas, from: <https://www.proteinatlas.org/ENSG00000145879-SPINK7/tissue>. (**B**) Representative images of IHC staining for Spink7 in murine esophagus (left panel) and skin (right panel) tissues. (**C**) Representative image of IHC staining for Spink7 in esophagus of KO mice. (**D**) Expression profile of Spink7 in different murine tissues detected by real-time RT-PCR (n=5). Blue column highlights esophagus and red column highlights back skin tissue. (**E**) Expression of Spink7 mRNA levels in day 1 and day 2 after wounding examined by real-time RT-PCR. n=4 per time point.

**Supplementary figure 2.** Double staining for Spink7 mRNA (FISH in red) and E-cadherin protein (IF in green) to determine Spink7 mRNA transcript in murine esophagus tissue. The white dashed line separates the epithelium from the lamina propria.

**Supplementary figure 3. SiRNAs against Spink7 knockdown Spink7 effectively in both in vitro cellular cultures and in vivo wounds. (A**) SiRNAs against Spink7 (si-61 and si-160) knockdown Spink7 protein levels by co-transfected with pCMV6-Spink7 plasmid in HEK-293 cells detected by western blot using Myc-tag antibody. (**B**) SiRNAs gels against Spink7 knockdown Spink7 mRNA levels in siRNA gels treated 7-day wounds examined by real-time RT-PCR. n=5 samples/group. **p*<0.05 versus si-NC treated group.

**Supplementary figure 4.** **Heatmap of microarray experiments and analysis of down-regulated genes through GO and KEGG.** (**A**) Heatmap of microarray experiments of 7-day wounds from both WT and KO mice. (**B**) Graph of top 10 biological process terms for GO analysis from down-regulated genes of microarray experiments. (**C**) Top 10 molecular function terms for GO analysis from down-regulated genes. (**D**) Top 10 pathway terms for KEGG analysis from down-regulated genes of microarray experiments.

**Supplementary figure 5. IHC staining for KLK5 and KLK7 in normal skin tissues from both WT and KO mice.** Top panel, KLK5; Bottom panel, KLK7.

**Supplementary figure 6. Inhibitory effect of** **CM containing Spink7 on the proteolytic activity of recombinant mouse KLK5.** Trypsin-like proteolytic activity measurement of recombinant mouse KLK5 (1.5 μg/ml) with addition of CM containing Spink7 protein or control. The experiments were performed in triplicate. **p*<0.05.

**Supplementary figure 7. Delayed skin wound healing of R-W-CI characterized by damped inflammatory response.** (**A**) Representative H&E sections for wounds on day 3 and 7 after wounding from both wounded-only and R-W-CI mice. Black arrows delineate wound edges. (**B**) High powder fields of H&E sections for day 3 (left panel) and day 7 (right panel) wounds from both wounded-only and R-W-CI mice. (**C**) Graphs of top 10 biological process terms for GO analysis from up-regulated (left panel) and down-regulated (right panel) genes of microarray experiments. Samples for microarray experiments were 3-day wound tissues from wounded-only and R-W-CI mice. (**D**) Top 10 pathway terms for KEGG analysis from up-regulated (left panel) and down-regulated (right panel) genes of microarray experiments. Red lines highlight peptidase related enrichments.

**Supplementary figure 8. IHC staining to detect expression of Spink7 in the wounds of R-W-CI model and evaluate the knockdown effect of siRNAs gel.** Top panel, expression of Spink7 in day 7 wounds of R-W-CI model. Bottom panel, expression of Spink7 in day 7 wounds of R-W-CI treated with siRNA gel.

**Supplementary figure 1**


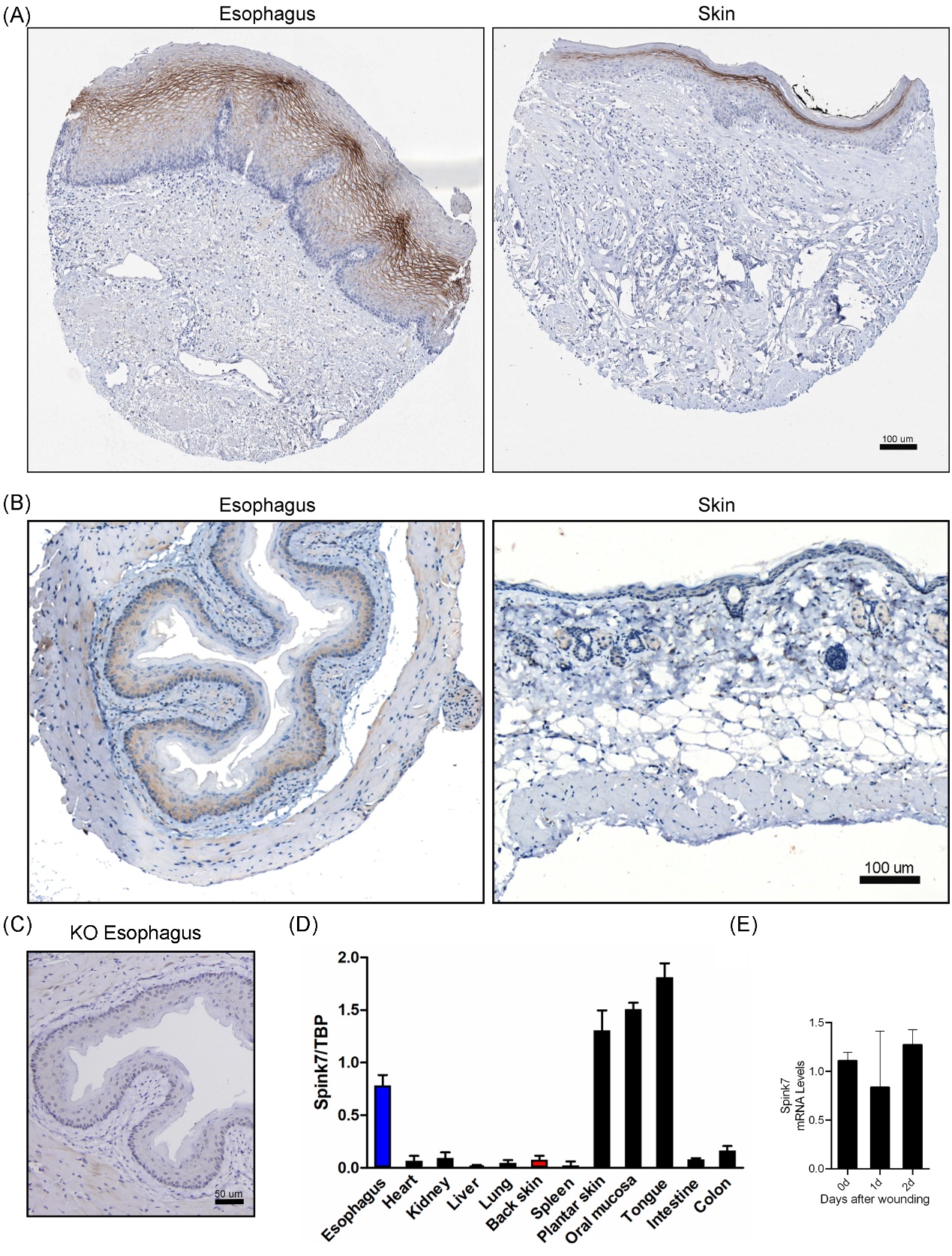


**Supplementary figure 2**


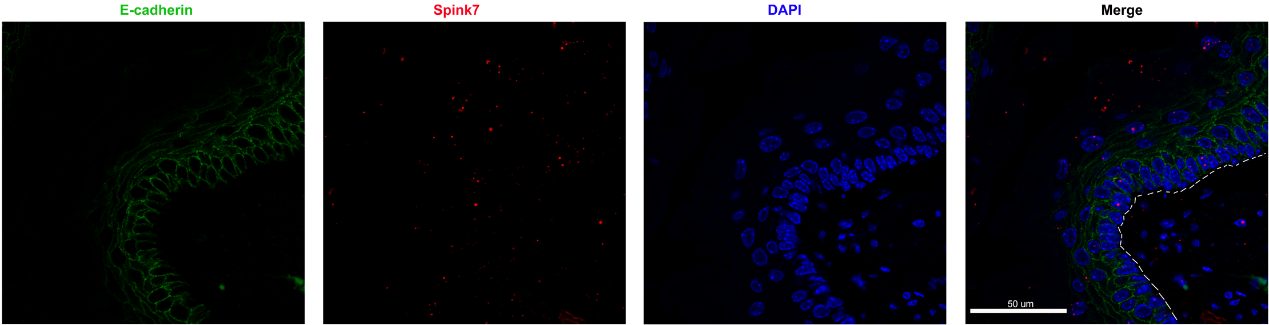


**Supplementary figure 3**


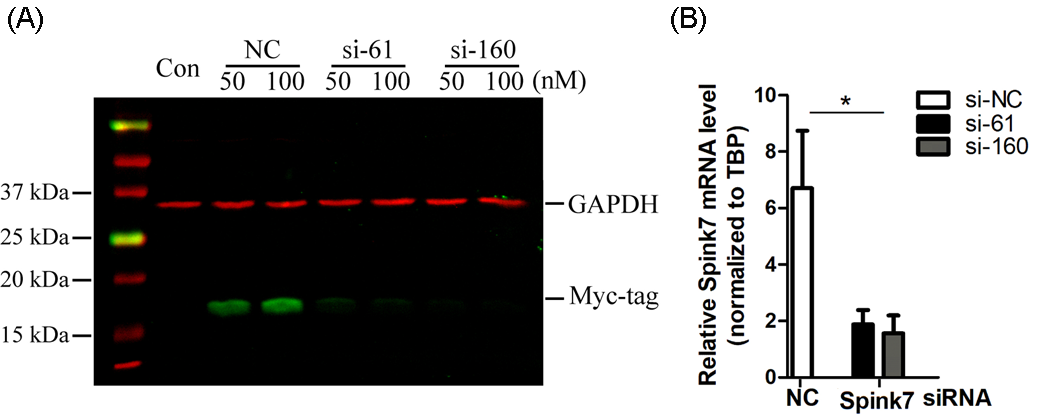


**Supplementary figure 4**


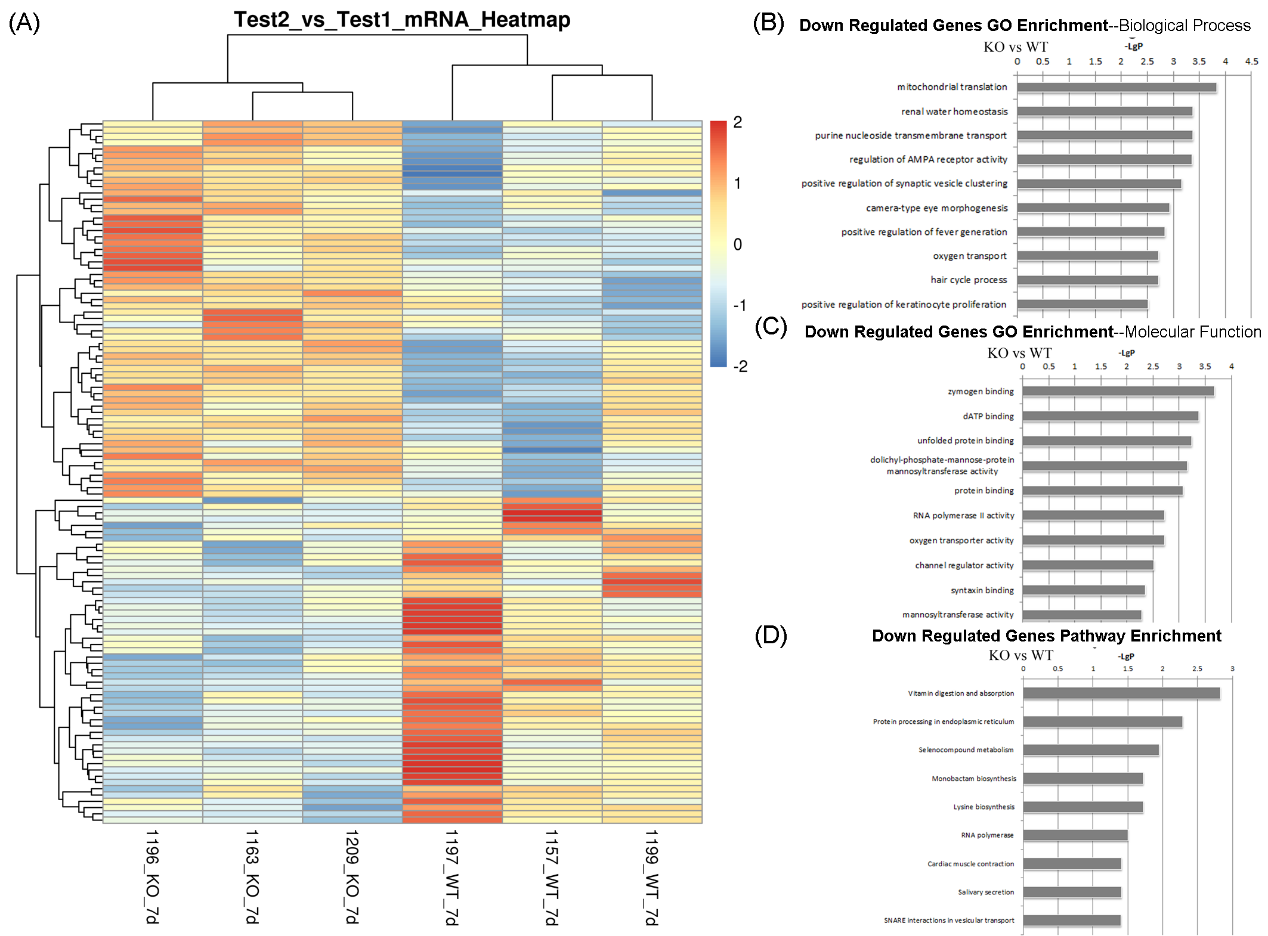


**Supplementary figure 5**


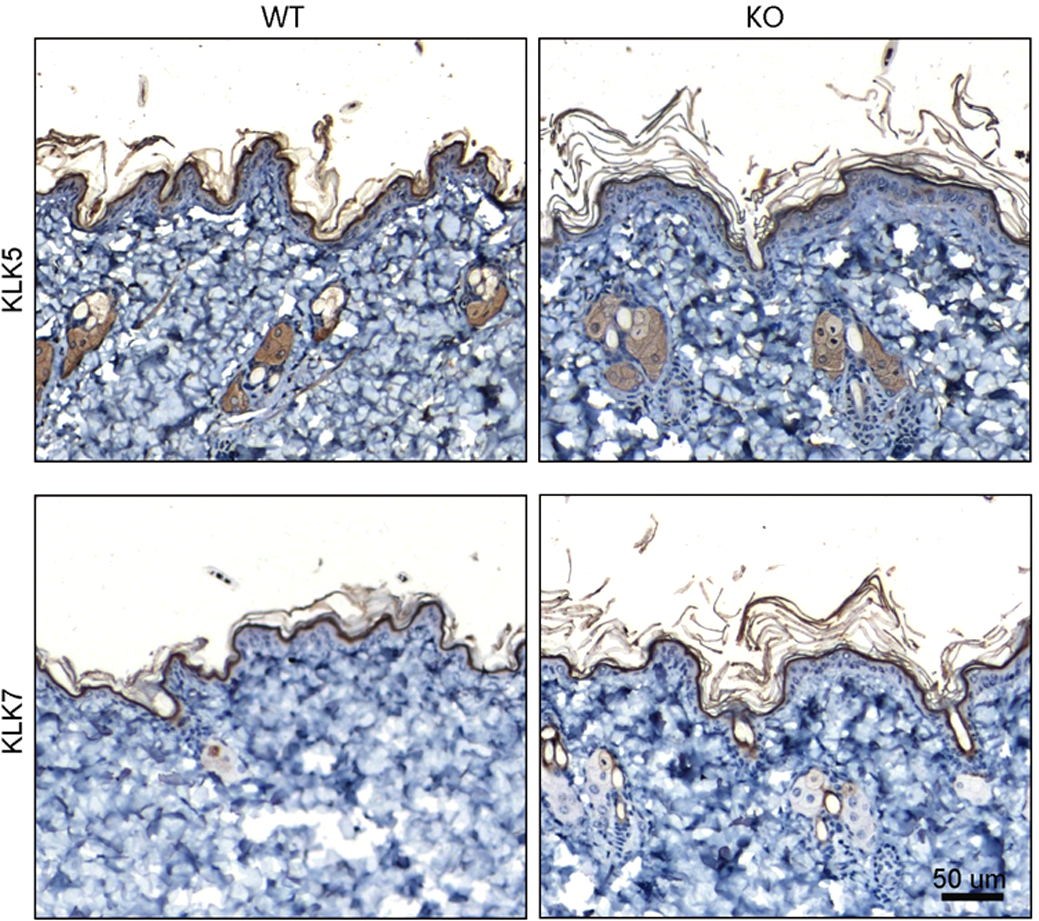


**Supplementary figure 6**


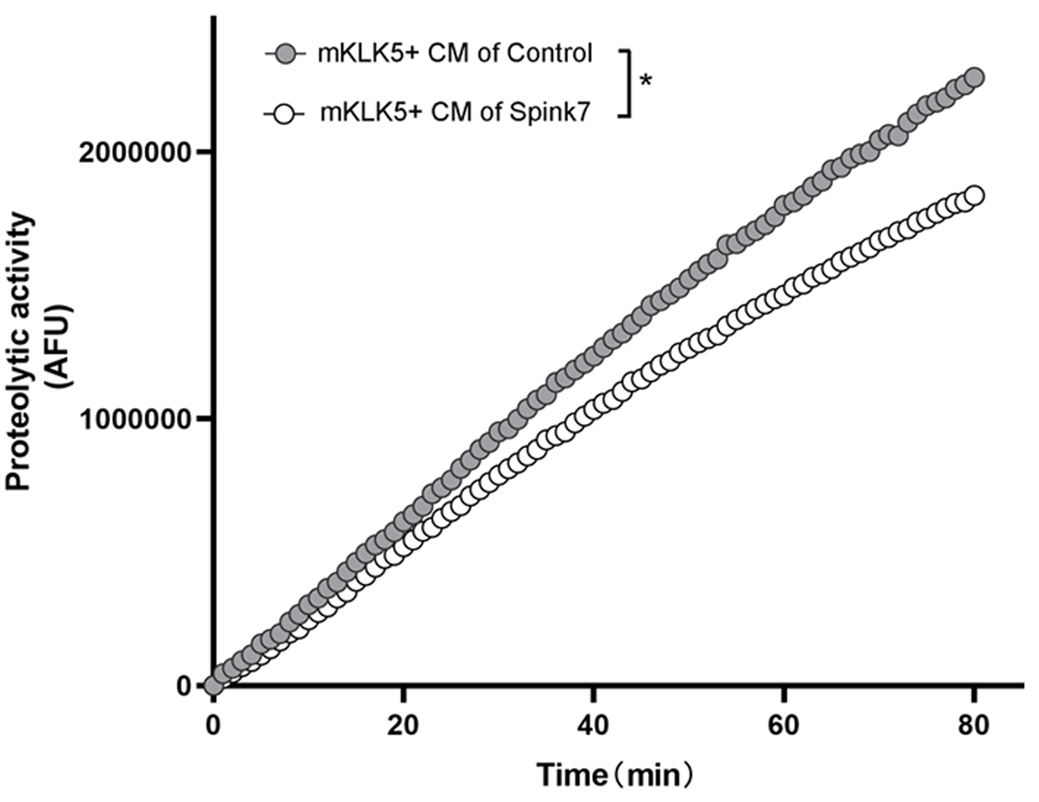


**Supplementary figure 7**


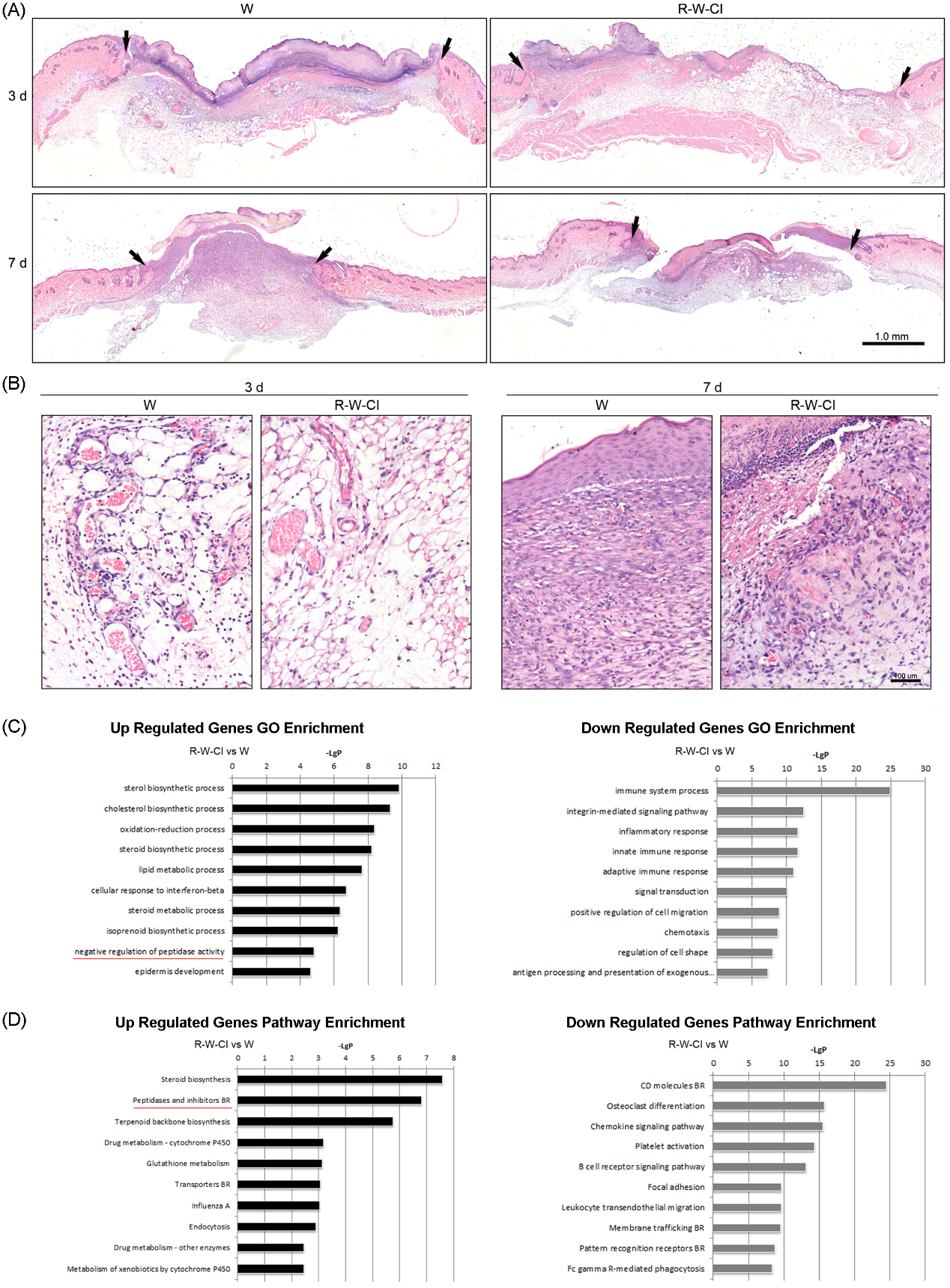


**Supplementary figure 8**


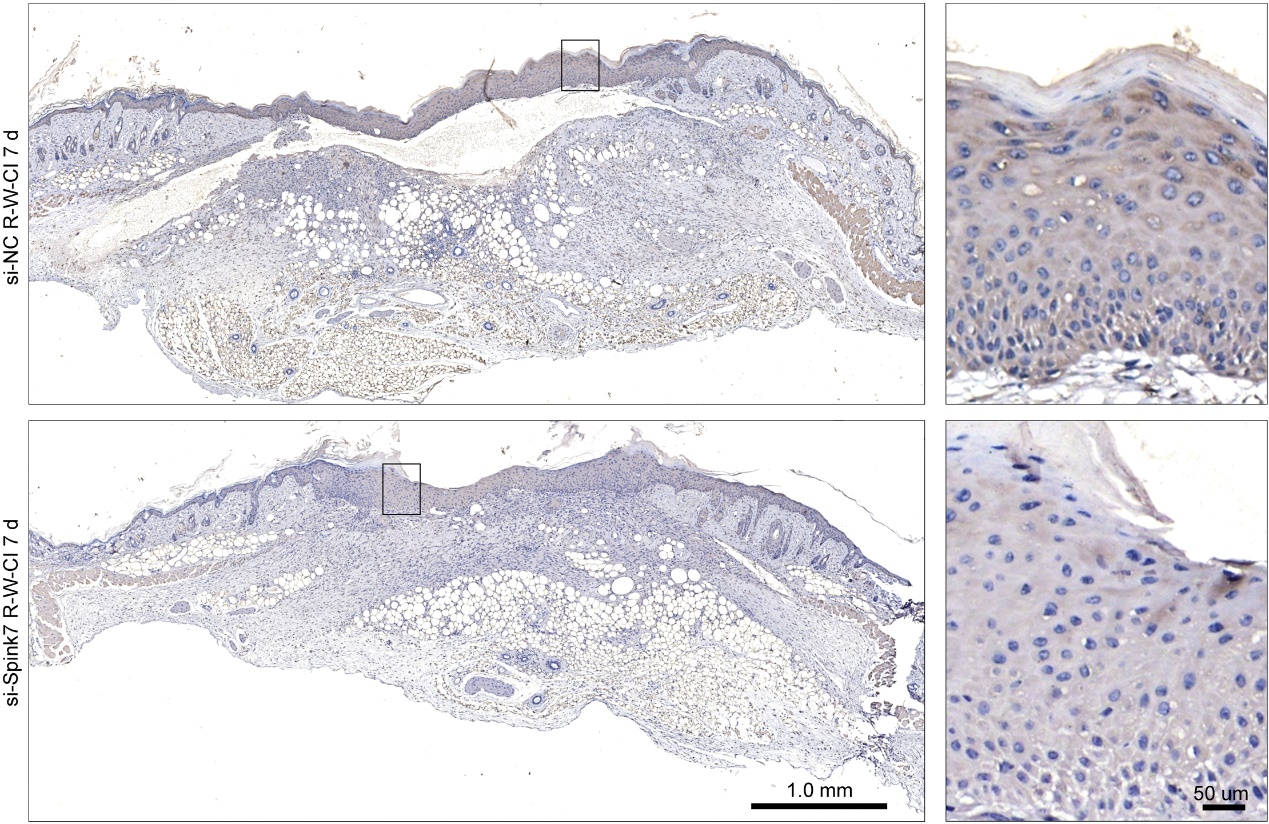

Supplement: Supplementary file 1 — Supporting Information [file CTM2-15-e70291-s002.docx]
