# Supplementary material for: The antiprotease Spink7 promotes inflammation resolution by modulating multiple proteases activities during wound healing
Source: Clin Transl Med. 2025 Mar 27;15(4):e70291. doi: 10.1002/ctm2.70291 (PMC11949503; doi:10.1002/ctm2.70291)
Supplement: Supplementary file 2 — Supporting Information [file CTM2-15-e70291-s001.docx]

**Supporting tables**

**Supplementary Table 1. Primers used in experiments**

| Primer sequences for qRT‒PCR |
| --- |

| TBP | Mouse | Forward | 5’‒ AAGGGAGAATCATGGACCAG ‒3’ |
| --- | --- | --- | --- |
|  |  | Reverse | 5’‒ CCGTAAGGCATCATTGGACT ‒3’ |
| GAPDH | Mouse | Forward | 5’‒ CCTCGTCCCGTAGACAAAATG‒3’ |
|  |  | Reverse | 5’‒ TCTCCACTTTGCCACTGCAA‒3’ |
| Spink7 | Mouse | Forward | 5’‒ TAGCCACCCTTCAGCAACAG ‒3’ |
|  |  | Reverse | 5’‒ ACTGGATTTTTCCATTGCTTCTCA ‒3’ |
| MPO | Mouse | Forward | 5’‒ GAGCCAGCTACCCGGTTCTC ‒3’ |
|  |  | Reverse | 5’‒ GGTCATTGGGTGGGATCTTG ‒3’ |
| IL1β | Mouse | Forward | 5’‒ TCTCGCAGCAGCACATCA ‒3’ |
|  |  | Reverse | 5’‒ CACACACCAGCAGGTTAT ‒3’ |
| IL1α | Mouse | Forward | 5’‒ TCTGCCATTGACCATCTC ‒3’ |
|  |  | Reverse | 5’‒ ATCTTCCCGTTGCTTGAC ‒3’ |
| IL6 | Mouse | Forward | 5’‒ TGGGAAATCGTGGAAATGAG ‒3’ |
|  |  | Reverse | 5’‒ CTCTGAAGGACTCTGGCTTTG ‒3’ |
| NOS2 | Mouse | Forward | 5’‒ TTCTGTGCTGTCCCAGTGAG ‒3’ |
|  |  | Reverse | 5’‒ TGAAGAAAACCCCTTGTGCT ‒3’ |
| TNFα | Mouse | Forward | 5’‒ CCCGGGCTCAGCCTCTTCTCATTC ‒3’ |
|  |  | Reverse | 5’‒ GGATCCGGTGGTTTGCTACGACGT ‒3’ |
| CXCL1 | Mouse | Forward | 5’‒ CTGGGATTCACCTCAAGAACATC ‒3’ |
|  |  | Reverse | 5’‒ CAGGGTCAAGGCAAGCCTC ‒3’ |
| CXCL2 | Mouse | Forward | 5’‒ CCTGCCAAGGGTTGACTTCA ‒3’ |
|  |  | Reverse | 5’‒ TTTTGACCGCCCTTGAGAGT ‒3’ |
| CCL3 | Mouse | Forward | 5’‒ CATATGGAGCTGACACCCCG ‒3’ |
|  |  | Reverse | 5’‒ GTCAGGAAAATGACACCTGGC ‒3’ |
| CCL4 | Mouse | Forward | 5’‒ CACCATGAAGCTCTGCGTGTC ‒3’ |
|  |  | Reverse | 5’‒GCAGGAAGTGGGAGGGTCAG‒3’ |
| Arg1 | Mouse | Forward | 5’‒ CAGAAGAATGGAAGAGTCAG ‒3’ |
|  |  | Reverse | 5’‒ CAGATATGCAGGGAGTCACC ‒3’ |
| Mrc1 | Mouse | Forward | 5’‒ CAGGTGTGGGCTCAGGTAGT ‒3’ |
|  |  | Reverse | 5’‒ TGGCATGTCCTGGAATGAT ‒3’ |
|  |  |  |  |

**Supplementary Table 2A. Antibodies used in IF and IHC analysis**

| Antibodies | Source | Catalog | Dilution |
| --- | --- | --- | --- |
| anti-MPO | Thermo | RB-373-A | 1:100 |
| anti-Ly6G | Biolegend | 127602 | 1:50 |
| anti-E-cadherin | CST | 3195S | 1:400 |
| anti-iNOS | Proteintech | 18985-1-AP | 1:200 |
| anti-CD206 | Proteintech | 18704-1-AP | 1:100 |
| anti-F4/80 | Biolegend | 123101 | 1:200 |
| anti-uPA | Proteintech | 17968-1-AP | 1:100 |
| anti-MMP2 | Abcam | ab86607 | 1:400 |
| anti-MMP9 | Abcam | ab228402 | 1:500 |
| anti-KLK5 | BOSTER | M03507 | 1:100 |
| anti-KLK7 | Abcam | ab254258 | 1:200 |
| Normal rabbit IgG antibody | Millipore | Cat#PP64 | RIP: 5μg; Dot Blot: 1:10000 |
| Anti-rabbit IgG, HRP-linked Antibody | CST | #7074S | WB: 1:2500 |
| Anti-rabbit IgG, HRP-linked Antibody | ZSGB-Bio | PV-6001 | IHC-p(ready for use) |
| Goat anti-rat IgG (H+L), Cross-adsorbed secondary antibody (Alexa Fluor^TM^ 488 Conjugate) | Thermo | A-11006 | IHC-P: 1:500 |
| Anti-rabbit IgG (H+L), F(ab')2 Fragment (Alexa Fluor®488 Conjugate) | CST | #4412S | IHC-P: 1:700 |
| Anti-rat IgG (H+L), F(ab')2 Fragment (Alexa Fluor^TM^ 594Conjugate) | Thermo | A-11007 | IHC-P: 1:500 |

**Supplementary Table 2B.** The primary antibodies used in Western blot analysis

| Antibodies | Source | Catalog | Dilution |
| --- | --- | --- | --- |
| anti-Arg1 | CST | 93668S | 1:1000 |
| anti-CD206 | CST | 24595S | 1:1000 |
| anti-iNOS | Abcam | ab178945 | 1:1000 |
| anti-Myc-tag | CST | 2276S | 1:1000 |
| anti-Tubulin | Abclonal | A12289 | 1:5000 |
| anti-β-actin | Beyotime | AF0003 | 1:1000 |
| anti-MMP2 | Abcam | ab86607 | 1:1000 |
| anti-MMP9 | Abcam | ab228402 | 1:1000 |
| anti-GAPDH | Beyotime | AF0006 | 1:1000 |
| anti-PAR2 | Abcam | ab180953 | 1:1000 |
| anti-TSLP | Abcam | ab188766 | 1:500 |
|  |  |  |  |
